# Development and validation of the symptom burden questionnaire for long covid (SBQ-LC): Rasch analysis

**DOI:** 10.1136/bmj-2022-070230

**Published:** 2022-04-27

**Authors:** Sarah E Hughes, Shamil Haroon, Anuradhaa Subramanian, Christel McMullan, Olalekan L Aiyegbusi, Grace M Turner, Louise Jackson, Elin Haf Davies, Chris Frost, Gary McNamara, Gary Price, Karen Matthews, Jennifer Camaradou, Jane Ormerod, Anita Walker, Melanie J Calvert

**Affiliations:** 1 Centre for Patient Reported Outcome Research, Institute of Applied Health Research, University of Birmingham, Birmingham, B15 2TT, UK; 2 National Institute for Health Research (NIHR) Applied Research Collaboration (ARC) West Midlands, Birmingham, UK; 3 Birmingham Health Partners Centre for Regulatory Science and Innovation, University of Birmingham, Birmingham, UK; 4 Institute of Applied Health Research, University of Birmingham, Birmingham, UK; 5 NIHR Surgical Reconstruction and Microbiology Research Centre, University Hospital Birmingham and University of Birmingham, UK; 6 NIHR Birmingham Biomedical Research Centre, University Hospital Birmingham and University of Birmingham, Birmingham, UK; 7 Centre for Trauma Science Research, University of Birmingham, Birmingham, UK; 8 Aparito, Wrexham, UK; 9 LongCOVIDSOS, Faringdon, UK; 10 Therapies for Long COVID (TLC) Study, University of Birmingham, Birmingham, UK; 11 COVID-END (COVID-19 Evidence synthesis Network to support Decision-making), McMaster University, Hamilton, ON, Canada; 12 Long COVID Scotland, Edinburgh, UK; 13 DEMAND Hub, University of Birmingham, Birmingham, UK; 14 Health Data Research UK, London, UK; 15 UK SPINE, University of Birmingham, Birmingham, UK

## Abstract

**Objective:**

To describe the development and validation of a novel patient reported outcome measure for symptom burden from long covid, the symptom burden questionnaire for long covid (SBQ-LC).

**Design:**

Multiphase, prospective mixed methods study.

**Setting:**

Remote data collection and social media channels in the United Kingdom, 14 April to 1 August 2021.

**Participants:**

13 adults (aged ≥18 years) with self-reported long covid and 10 clinicians evaluated content validity. 274 adults with long covid field tested the draft questionnaire.

**Main outcome measures:**

Published systematic reviews informed development of SBQ-LC’s conceptual framework and initial item pool. Thematic analysis of transcripts from cognitive debriefing interviews and online clinician surveys established content validity. Consensus discussions with the patient and public involvement group of the Therapies for Long COVID in non-hospitalised individuals: From symptoms, patient reported outcomes and immunology to targeted therapies (TLC Study) confirmed face validity. Rasch analysis of field test data guided item and scale refinement and provided initial evidence of the SBQ-LC’s measurement properties.

**Results:**

SBQ-LC (version 1.0) is a modular instrument measuring patient reported outcomes and is composed of 17 independent scales with promising psychometric properties. Respondents rate their symptom burden during the past seven days using a dichotomous response or 4 point rating scale. Each scale provides coverage of a different symptom domain and returns a summed raw score that can be transformed to a linear (0-100) score. Higher scores represent higher symptom burden. After rating scale refinement and item reduction, all scales satisfied the Rasch model requirements for unidimensionality (principal component analysis of residuals: first residual contrast values <2.00 eigenvalue units) and item fit (outfit mean square values within 0.5 -1.5 logits). Rating scale categories were ordered with acceptable category fit statistics (outfit mean square values <2.0 logits). 14 item pairs had evidence of local dependency (residual correlation values >0.4). Across the 17 scales, person reliability ranged from 0.34 to 0.87, person separation ranged from 0.71 to 2.56, item separation ranged from 1.34 to 13.86, and internal consistency reliability (Cronbach’s alpha) ranged from 0.56 to 0.91.

**Conclusions:**

SBQ-LC (version 1.0) is a comprehensive patient reported outcome instrument developed using modern psychometric methods. It measures symptoms of long covid important to people with lived experience of the condition and may be used to evaluate the impact of interventions and inform best practice in clinical management.

## Introduction

Since the emergence of SARS-CoV-2 in 2019, the covid-19 pandemic has resulted in more than 450 million infections and more than six million deaths worldwide.^1^ Although infection is mild and short lived for many people, a proportion continue to experience or go on to develop symptoms that persist beyond the acute phase of infection. These persistent symptoms are known collectively as post-acute sequelae of covid-19, post-acute covid-19, post-covid-19 syndrome, post-covid-19 condition, or long covid.^2^ ^3^

Symptom burden can be defined as the “subjective, quantifiable prevalence, frequency, and severity of symptoms placing a physiologic burden on patients and producing multiple negative, physical, and emotional patient responses.”^4^ The symptoms reported by those with long covid are heterogenous and can affect multiple organ systems, with fatigue, dyspnoea, and impaired concentration among the most prevalent symptoms.^5^ ^6^ ^7^ Symptoms may be persistent, cyclical, or episodic and can pose a substantial burden for affected individuals, with negative consequences for work capability, functioning, and quality of life.^8^ ^9^ ^10^ There is a growing body of research on the prevalence, incidence, co-occurrence, and persistence of the signs and symptoms of long covid.^5^ ^6^ ^8^ ^11^ ^12^ ^13^ These data have largely been collected using bespoke, cross sectional survey tools due, in part, to the limited availability of condition specific, validated self-report instruments.^14^

Patient reported outcomes are measures of health reported directly by patients without amendment or interpretation by clinicians or anyone else.^15^ Validated instruments measuring patient reported outcomes developed specifically for long covid that address the complex, multifactorial nature of the condition are needed urgently to further the understanding of long covid symptoms and underlying pathophysiology, support best practice in the clinical management of patients, and evaluate the safety, effectiveness, acceptability, and tolerability of interventions.^16^ ^17^ ^18^ Validated instruments to measure patient reported outcomes have recently been developed to measure the global impact of long covid, and several unvalidated screening tools, surveys, and questionnaires are also available.^19^ ^20^ However, individuals living with long covid have suggested that existing self-report measures fail to capture the breadth of experienced symptoms.^10^ ^21^ ^22^ To address the need for a comprehensive measure of self-reported symptom burden specific to long covid, we used Rasch analysis to develop and validate, in accordance with US Food and Drug Administration guidance, a novel instrument measuring patient reported outcomes, the symptom burden questionnaire for long covid (SBQ-LC).^15^ ^23^

## Methods

### Setting and study design

**Fig 1:**
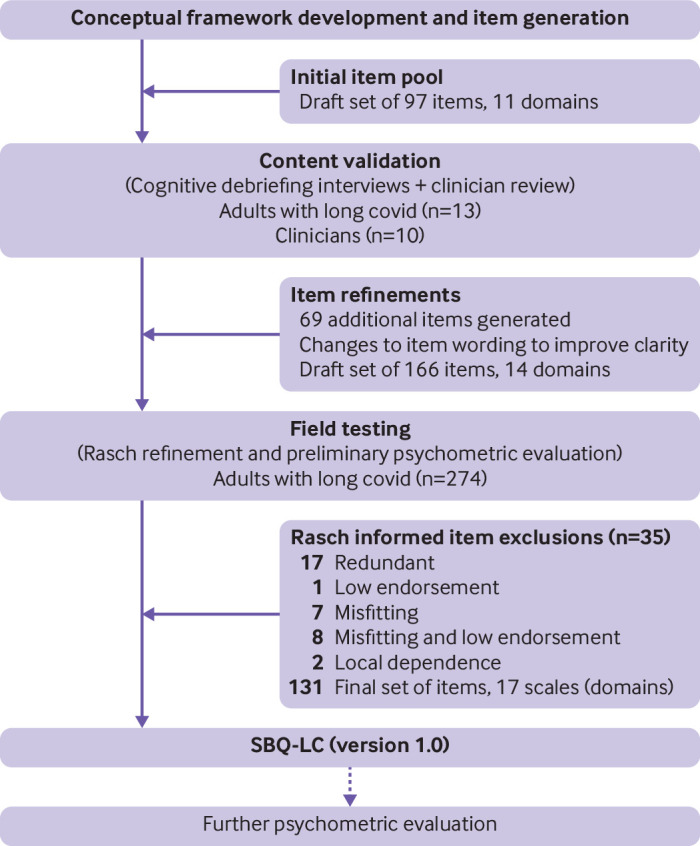
Development of symptom burden questionnaire for long covid (SBQ-LC)

This multiphase, prospective mixed methods study (fig 1) was nested within the Therapies for Long COVID in non-hospitalised individuals: From symptoms, patient reported outcomes and immunology to target therapies (TLC) Study.^24^ The study took place from 14 April to 1 August 2021.

### Study population

Content validation was undertaken with adults with long covid recruited from the TLC study’s patient and public involvement (PPI) group and clinicians recruited from the TLC study and long covid research studies based in the UK. The field test population included adults with self-reported long covid. Participants were aged 18 years or older who could self-complete SBQ-LC in English. No exclusion criteria relating to duration of long covid symptoms, hospital admissions for SARS CoV-2 infection, or vaccination status were applied. A minimum sample size of 250 respondents was prespecified for field testing. In Rasch analysis, a sample of 250 respondents provides 99% confidence that item calibrations and person measures are stable within ±0.50 logits.^25^

### Symptom coverage and existing patient reported outcomes

The conceptual framework underpinning SBQ-LC was developed from systematic literature reviews of long covid symptoms.^5^ ^26^ Existing symptom measures (n=6) with good face validity in the context of long covid were reviewed to establish whether a new instrument for symptom burden that measured patient reported outcomes was needed.^20^ ^27^ ^28^ ^29^ ^30^ ^31^ When mapped to the conceptual framework, symptom coverage of these instruments ranged from 27.0% to 60.3%: mean 34.5% (standard deviation 16.2%). Supplementary table S1 presents the concept coverage matrix mapping symptom coverage of the candidate instruments to the conceptual framework. The finding from this mapping suggested that complete coverage of long covid symptoms could not be guaranteed using existing measures, providing justification for the development of SBQ-LC.

### Study procedures

#### Content validation

Content validation involved an online clinician survey to explore item relevance and clarity and identify symptoms of clinical concern, and cognitive debriefing interviews with adults with long covid to ascertain the relevance, comprehensiveness, comprehensibility, and acceptability of SBQ-LC’s items for the target population. The clinician survey (supplementary file S1) was administered using the survey software application SmartSurvey.^32^ A content validity index value was calculated for each item (item-content validity index) as the proportion of clinicians who rated the item as relevant adjusted for chance agreement (modified κ). A modified κ value in the range of 0.4-0.59 was considered as fair content validity, 0.60-0.74 as good, and ≥0.74 as excellent.^33^ We used the item-content validity index values to identify item candidates requiring in-depth exploration of relevance and comprehensibility with long covid patients.

Cognitive debriefing interviews took place through videoconferencing and were recorded. Verbatim transcripts of the interview recordings, field notes, and free text comments from the clinician survey were analysed qualitatively using thematic analysis and a prespecified framework to identify problems with the relevance, comprehensiveness, clarity, and acceptability of the items.^34^ ^35^ Participants with lived experience of long covid were asked to identify additional symptoms not present in the initial item pool. Findings informed revisions, which were tracked for each item using an Excel spreadsheet. A draft version of SBQ-LC was constructed and sent forward for field testing.

#### Field testing

The Atom5 platform (Apartio, Wrexham, UK) is a regulated (ISO13485, ISO/IEC 27001:2013 Accreditation, FDA CFR21 Part 11 compliant) software platform that provides remote data collection and real time patient monitoring through a smartphone application and integration with wearable devices.^36^ The draft SBQ-LC, the EQ-5D-5L as a measure of health related quality of life, and a demographic questionnaire were programmed onto Atom5 for delivery.^37^ ^38^ Participants were recruited through social media advertisements posted on Twitter and Facebook by the study team and through other support group platforms and website registrations in collaboration with long covid support groups based in the UK. Interested individuals connected via a URL to a study specific website where they could read detailed information about the study, provide informed consent, and download the Atom5 app to their mobile device (smartphone or tablet). Participants accessed the questionnaires by way of a unique QR code. Once the completed questionnaires were submitted, participants could delete the app from their phone. We securely downloaded the anonymised field test data from Atom5 for analysis.

### Statistical analyses

STATA (version 16) was used to clean and prepare the data, and for descriptive data analyses. We conducted Rasch analysis on the field test data to refine SBQ-LC (ie, item reduction) and assess its scaling properties. A Rasch analysis is the formal evaluation of an instrument that measures patient reported outcomes against the Rasch measurement model. The Rasch model is a mathematical ideal that specifies a set of criteria for the construction of interval level measures from ordinal data.^39^ It is a probabilistic model, which specifies that an individual’s response to an item is only governed by the individual and the location of the item on a shared scale measuring the latent trait. The probability that a person will endorse an item is a logistic function of the difference between an individual’s trait level (expressed as person ability) and the amount of trait expressed by the item (expressed as item difficulty).^40^ ^41^

Rasch analysis enabled SBQ-LC to be constructed as a modular instrument measuring patient reported outcomes (ie, a multi-domain item bank) with linear, interval level measurement properties. These properties render Rasch developed patient reported outcomes suitable for use with individual patients as well as for group level comparisons, permit direct comparisons of scores across domains, and facilitate the construction of alternative test formats (ie, short forms and computer adaptive tests).^42^

Rasch analyses were carried out using Winsteps software (version 5.0.5) and the partial credit model for polytomous data.^43^ We selected the partial credit model because the question wording and rating scale categories varied across items. Joint maximum likelihood estimation in Winsteps enabled parameter estimation when data were missing. Misfitting response patterns (eg, arising from respondents guessing or other unexpected behaviour) have been shown to result in biased item estimates with detrimental impacts for model fit.^44^ ^45^ Therefore, as is customary in Rasch analysis, we appraised person fit statistics, iteratively removed individuals with misfitting response patterns (ie, outfit mean square values >2.0 logits), and re-estimated item parameters until evidence of item parameter stability was observed.^40^ ^44^

Rating scale functioning for individual items was assessed against several criteria: all items oriented in the same direction as a check for data entry errors (ie, appraisal of point measure correlations); average category measures advance (ie, higher categories reflect higher measures); category outfit mean square values ≤2.0 logits (ie, as an indicator of unexpected randomness in the model); and each category endorsed by a minimum of 10 respondents.^46^ If an item’s rating scale failed to meet these criteria, we combined adjacent categories or removed the item. Category probability curves provided a graphical representation as further evidence of rating scale functioning.

To confirm model fit, we completed Rasch analyses (including appraisal of unidimensionality, local independence, and individual item fit statistics) iteratively as items were removed or grouped to create new scales. We also evaluated person reliability and separation indices and scale-to-sample targeting. Targeting examines the correspondence between items and individuals, and, for a well targeted scale, the items in a scale should be spaced evenly across a reasonable range of the scale and correspond to the range of the construct experienced by the sample.^40^ Person reliability examines the reproducibility of relative measure location, and person separation provides a measure of the number of distinct levels of person ability (symptom burden) that can be distinguished by a scale.^47^ For each scale we computed Cronbach’s alpha as a measure of internal consistency reliability.^48^ Box 1 describes the parameters evaluated in the development and validation of SBQ-LC, along with acceptability criteria. EQ-5D-5L values were generated following guidance from the National Institute for Health and Care Excellence.^49^ Valuation was undertaken using the crosswalk method to the EQ-5D-3L value set.^50^ We compared against published data on population norms. This analysis was, however, exploratory only and therefore a representative study would be needed to comprehensively analyse the effects on health related quality of life associated with long covid.

Box 1 **Rasch measurement properties, definition or aim of evaluation, and acceptability criteria**

**Valid measurement model**

To identify a set of items that effectively measure the target construct of symptom burden in people with long covid (ie, fulfil the axioms of fundamental measurement permitting the construction of interval level scales)

*Acceptability criteria for fit*

Unidimensionality: Principal component analysis of residuals, highest eigenvalue of first residual contrast <2.0; disattenuated correlations >0.70
Local item independence: Residual correlation <0.40
Fit statistics: Outfit/infit mean square values within 0.5-1.5 logits
Point measure correlations >0.40
Rating scales: Scale oriented with latent variable, categories advance monotonically, category fit statistics: Outfit mean square values <2.0 logits, uniform category endorsement

**Rasch reliability**

Rasch based reliability is the share of true variance of the total observed variance of the measure; person separation index is the number of distinct levels of person ability (symptom burden) that can be identified by the measure

*Acceptability criteria for fit*

Person reliability: r≥0.70 acceptable; r≥0.80 good; r≥0.90 excellent
Person separation index: 1.5-2.0 acceptable level; 2.0-3.0 good level; ≥3.0 excellent level

**Internal consistency reliability**

Extent to which items comprising a scale measure the same construct (degree of homogeneity or relatedness among items of a scale)

*Acceptability criteria for fit*

Cronbach’s alpha range ≥0.70

**Targeting**

Extent to which the range of the variable measured by the scale matches the range of that variable in the study sample

*Acceptability criteria for fit*

Item person map
Acceptable targeting shown by close correspondence of the person mean with the item mean for a scale (±1.0 logits from the mean of zero)

### Patient and public involvement

The TLC study PPI group was established in line with guidance from the National Institute for Health Research improving inclusion of under-served groups in clinical research (INCLUDE) project.^51^ Members of the TLC study PPI group and representatives from UK based long covid support groups were involved from the outset in the development of the study design, recruitment strategy, and all participant facing materials. Field test participants were recruited from long covid patient support groups identified on social media channels. PPI members reviewed and provided critical feedback during the drafting of the manuscript. We will work with PPI members to disseminate the study results to relevant patient and public communities.

## Results

### Item development and content validation

An initial pool of 97 items was constructed, guided by the conceptual framework developed from the published literature. The clinicians’ review (n=10) of the item pool informed changes to the wording of items to improve clarity. Content validity indices were calculated for each item (item-content validity index) based on clinician ratings of relevance and used to identify items requiring further investigation of relevancy during cognitive debriefing. Item-content validity index values ranged from 0.4 to 1.0, with 115 (94%) of the draft items rated as good or as excellent (supplementary table S2). Content validity was confirmed by 13 people with lived experience of long covid in two rounds of cognitive debriefing interviews. All participants were white and ranged in age from 20 to 60 years. Ten participants (77%) were women. Cognitive debriefing identified gaps in symptom coverage, resulting in the generation of 69 new items. Findings also guided the design of the rating scale layout in Atom5 and confirmed patient preferences for response category labels. Thematic analysis classified problems with draft items’ relevance, comprehensiveness, clarity, and acceptability. Supplementary table S3 presents key themes, together with exemplar quotations, from the thematic analysis.

The draft SBQ-LC included 166 items (155 symptoms and 11 interference items) and an a priori theoretical domain classification comprised of 14 domains, each constructed as an independent scale. Items utilised a seven day recall period, and burden was measured using a dichotomous response (yes or no) or a 5 point rating scale measuring either severity, frequency, or interference. Higher scores represented greater symptom burden. Commonly experienced symptoms were presented earlier in each scale, and potentially sensitive items (eg, self-harm) were positioned in the middle or at the end of a scale. Neutral wording ensured items were not phrased as leading questions. Response scales with empirical evidence of their use in validated instruments measuring patient reported outcomes reinforced the rigor of SBQ-LC’s design.^52^ To confirm face validity, we held a virtual meeting with the TLC Study’s PPI group in May 2021 to obtain consensus on the utility, acceptability, and format of SBQ-LC.

### Readability

Readability, measured using the Flesch-Kincaid reading grade level test and the Simple Measure of Gobbledygook (SMOG) index score, was calculated using the web based application Readable.^53^ ^54^ ^55^ The American Medical Association and the National Institutes of Health recommend that the readability of patient materials should not exceed a sixth grade reading level (Flesh-Kincaid score 6.0).^56^ SBQ-LC’s Flesch-Kincaid reading grade level score was 5.33 and SMOG index score was 8.27. Text with a SMOG index score ranging between 7 and 9 would be understood by 93% of adults in the UK.^57^

### Field testing

#### Participants

Over a two week period in June 2021, 906 questionnaires were delivered and 330 responses were received (response rate 36%). Fifty six submissions were incomplete and excluded from the analyses. The final sample included 274 complete responses (completion rate 83%). The age of respondents ranged from 21 to 70 years, with a mean age of 45.0 (standard deviation 10.0) years. The sample included 240 (88%) female respondents, and 253 (92%) respondents were white. All respondents (100%) had self-reported covid-19, with half the field test sample (n=150, 55%) being infected during the first wave of the pandemic, defined as March to May 2020 in England.^58^ One hundred and twenty nine (47%) respondents reported having a positive polymerase chain reaction test result for SARS CoV-2, and 22 (8%) reported having a positive lateral flow test result. Owing to limited availability of testing early in the pandemic, not all participants were tested for SARS-CoV-2. Eleven respondents (4%) had been admitted to hospital with covid-19. Seventy (25%) respondents had received one dose of a covid-19 vaccine and 187 (68%) had received two doses. In total, 153 (56%) respondents were in either full time or part time employment (table 1). Exploratory analysis showed that EQ-5D-5L values (mean score 0.490 (standard deviation 0.253)) were lower than UK population norms reported in published studies and suggested that further research is needed to evaluate the impacts of long covid on health related quality of life—for example, the following EQ-5D values were reported in a recent study for the indicated age groups in England, 25-34: 0.919; 35-44: 0.893; 45-54: 0.855; 55-64: 0.810; 65-74: 0.773; and ≥75: 0.703).^59^ Overall, 214 respondents (78%) reported one or more comorbidities (table 2).

**Table 1:** Personal characteristics of field test sample. Values are numbers (percentages) of participants unless stated otherwise

Characteristics	Respondents (n=274)
Age (years):	
Mean (SD)*; range	45.0 (10.0); 21-70
Sex:	
Female	240 (88)
Male	34 (12)
Ethnicity:	
White	253 (92)
Asian or Asian British	7 (3)
Black, African, Caribbean, or black British	3 (1)
Mixed or multiple ethnic groups	11 (4)
Other ethnic group	0 (0)
Occupational status:	
Employed full time	114 (42)
Employed but currently not working	51 (19)
Employed part time	39 (14)
Furloughed	7 (3)
Retired	6 (2)
Caregiver	3 (1)
In full time education	3 (1)
Voluntary work	3 (1)
Unemployed	20 (7)
Other	28 (10)
Month and year of SARS-Co-V-2 infection:	
December 2019	3 (1)
January-December 2020	233 (85)
January-May 2021	38 (14)
Positive PCR test result for SARS-Co-V-2 infection†:	
Yes	129 (47)
No	137 (50)
Do not know	8 (3)
Positive lateral flow test result for SARS-Co-V-2 infection†:	
Yes	22 (8)
No	227 (83)
Do not know	25 (9)
Admitted to hospital for SAR-Co-V-2 infection:	
Yes	11 (4)
No	263 (96)
Admitted to ICU for SARS-Co-V-2 infection:	
Yes	0 (0)
No	274 (100)
Attended hospital emergency department for SARS-Co-V-2 infection:	
Yes	111 (41)
No	163 (59)
Vaccine status:	
Two doses	187 (68)
One dose	70 (25)
No dose	17 (6)
Received shielding letter from UK government‡:	
Yes	12 (4)
No	262 (96)
Care home resident:	
Yes	3 (1)
No	271 (99)
Mean (SD) EQ-5D-5L utility score	0.490 (0.253)

SD=standard deviation; PCR=polymerase chain reaction; ICU=intensive care unit; EQ-5D-5L=health related quality of life instrument.

* n=263 respondents.

† Owing to difficulties accessing PCR and lateral flow tests in the early weeks of the pandemic, not all participants had access to testing.

‡ Letter to indicate clinical vulnerability requiring enhanced social distancing.

**Table 2:** Number and types of self-reported comorbidities in field test sample

Comorbidities	No (%) of respondents (n=274)
No of comorbidities:	
0	60 (22)
1	77 (28)
2	54 (20)
3	38 (14)
≥4	45 (16)
Comorbidities:	
Anxiety	93 (34)
Asthma	65 (24)
Depression	61 (22)
Irritable bowel syndrome	55 (20)
Other	53 (19)
Back or neck pain, or both	49 (18)
Hypertension	30 (11)
Chronic fatigue syndrome	29 (11)
Osteoarthritis	18 (7)
Polycystic ovary syndrome	11 (4)
Diabetes	8 (2.9)
Rheumatoid arthritis	5 (1.8)
Coeliac disease	4 (1.5)
Chronic obstructive pulmonary disease	3 (1)
Inflammatory bowel disease	3 (1)
Spleen	3 (1)
Stroke or transient ischaemic attack	3 (1)
Cancer	2 (0.7)
Epilepsy	2 (0.7)
Heart disease	2 (0.7)
Immunosuppression treatment	2 (0.7)
Osteoporosis	2 (0.7)
Cystic fibrosis	1 (0.4)
Kidney disease	1 (0.4)
Liver disease	1 (0.4)
Severe combined immunodeficiency	1 (0.4)
Sickle cell anaemia	1 (0.4)
Atrial fibrillation	0 (0)
Dementia	0 (0)
Down’s syndrome	0 (0)
Multiple sclerosis	0 (0)
Parkinson’s disease	0 (0)
Transplant recipient	0 (0)

#### Assessment of rating scale functioning, item fit, and scale refinement

To assess rating scale functioning we examined the relevant Winsteps output tables and item category probability curves for 166 items. Appraisal of category endorsement revealed the presence of floor effects and positively skewed scoring distributions. To deal with non-uniform category distribution, we collapsed the 5 point rating scale to either a dichotomous response or a 4 point rating scale (0=none, 1=mild, 2=moderate, 3=severe; 0=never, 1=rarely, 2=sometimes, 3=always; 0=not at all, 1=very little, 2=somewhat, 3=severely). After adjustment of the rating scale and removal of 35 items (figure 1 shows the iterative process of item reduction together with reasons for removal), point-measure correlations were positive (range 0.23-0.92), categories were ordered, and category fit statistics were productive for measurement (outfit mean square values <2.0 logits) for all items. Category distribution remained positively skewed overall (mean skewness 1.44, mean standard error 0.15 (range −2.30-11.64)) and disordered thresholds were observed for 52 (40%) items (supplementary figure S1). Threshold disordering, indicative of low category endorsement, is only considered a cause for concern when category disordering is also observed.^43^ Consequently, no further items were removed. We systematically grouped the remaining 131 items to construct scales that were clinically sensible and satisfied the Rasch model requirements of unidimensionality, item fit, and local independence.

#### Calibration of final SBQ-LC

**Fig 2:**
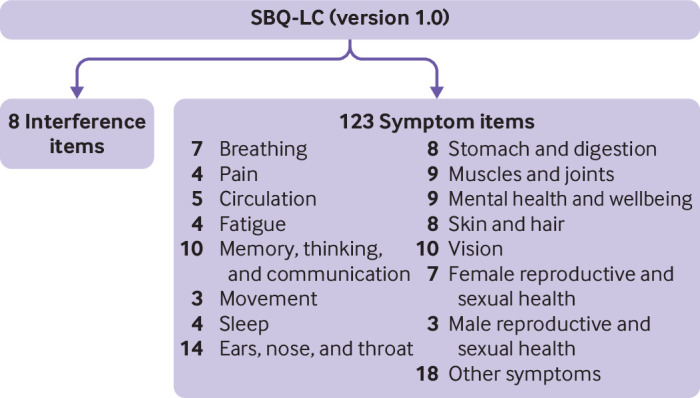
Conceptual framework showing scales (domains) of symptom burden questionnaire for long covid (SBQ-LC, version 1.0)

After optimisation of the response scale, we performed Rasch analyses to report the psychometric properties of the finalised SBQ-LC (version 1.0). SBQ-LC (version 1.0) is composed of 17 independent scales (fig 2). Supplementary table S4 presents the items included in each of SBQ-LC’s scales. To illustrate the respondent instructions, item wording, and response scales, in supplementary file S2 we present the SBQ-LC breathing scale as an exemplar. Table 3 presents the Rasch based statistics for each of SBQ-LC’s scales. All scales met the Rasch model criteria for unidimensionality and item fit. The first residual contrast values from principal component analyses of the residuals ranged from 1.46 to 2.03 eigenvalues. No serious misfit was identified. Item infit mean square values ranged from 0.67 to 1.32 logits and outfit mean square values ranged from 0.44 to 1.53 logits. Fourteen item pairs across eight scales showed local item dependency, with residual correlation values >0.4 (range 0.44-0.88). In practical terms, a degree of local dependency is always observed in empirical data; therefore, it is necessary to consider the implications for content validity before proceeding with item removal.^60^ After qualitative appraisal of the 14 dependent pairs, we retained all items to ensure comprehensive symptom coverage.

**Table 3:** Summary of scale level Rasch based psychometric properties for symptom burden questionnaire for long covid (SBQ-LC, version 1.0) in 274 adults with long covid

Scale (symptom domain)	No of items	Misfitting items	Misfitting persons removed (%)	Mean person ability (logits)	PCA eigenvalue (first contrast)^*^	Dependent item pairs	Item separation	Item reliability	Person separation†	Person reliability‡	Internal consistency reliability§
Breathing	7	0	31 (11.3)	−1.95 (SE 0.99)	1.72	0	7.68	0.98	1.8	0.76	0.84
Circulation	5	0	17 (6.2)	−1.16 (SE 0.98)	1.58	0	7.43	0.98	1.1	0.55	0.63
Fatigue	4	0	33 (12.0)	3.98 (SE 1.45)	1.46	1	1.34	0.64	1.78	0.76	0.91
Memory, thinking, and communication	10	0	12 (4.38)	−1.04 (SE 0.54)	1.72	0	13.86	0.99	2.56	0.87	0.9
Sleep	4	0	31(11.3)	−0.10 (SE 0.78)	1.62	2	13.44	0.99	1.67	0.74	0.56
Movement	3	0	31 (11.3)	−6.27 (SE 2.06)	1.57	3	11.23	0.99	2.08	0.81	0.86
Muscles and joints	9	0	23 (8.39)	−0.85 (SE 0.54)	1.68	0	7.38	0.98	1.68	0.73	0.84
Skin and hair	8	0	26 (9.49)	−2.38 (SE 0.92)	1.55	0	4.6	0.95	0.71	0.34	0.68
Eyes	10	0	12 (4.38)	−1.21 (SE 0.72)	1.85	0	4.28	0.95	1.08	0.54	0.72
Ears, nose, and throat	14	0	11 (4.01)	−1.25 (SE 0.49)	1.96	1	3.39	0.92	1.22	0.6	0.8
Stomach and digestion	8	1	23 (8.39)	−1.52 (SE 0.70)	1.6	0	6.19	0.97	0.95	0.48	0.7
Mental health and wellbeing	9	0	25 (9.12)	−0.70 (SE 0.52)	1.66	0	8.71	0.99	1.78	0.76	0.82
Female reproductive and sexual health	7	0	27 (9.85)	−2.07 (SE 1.05)	1.99	2	5.52	0.97	0.99	0.5	0.59
Male reproductive and sexual health	3	0	1 (3.03)	−1.48 (SE 1.98)	2.03	2	2.27	0.84	0.79	0.38	0.6
Pain	4	0	25 (9.12)	−1.53 (SE 1.05)	1.75	2	13.06	0.99	1.62	0.72	0.77
Other symptoms	18	0	22 (8.03)	−1.53 (SE 0.43)	1.67	1	5.5	0.97	1.33	0.64	0.79
Interference	8	0	21 (7.66)	2.35 (SE 0.89)	1.95	0	11.21	0.99	2.13	0.82	0.89

SE=standard error.

* Unidimensionality: principal component analyses (PCA) of residuals eigenvalue of first residual contrast <2.0.

† Person separation: ≥1.50 acceptable, ≥2.00 good, ≥3.00 excellent.

‡ Rasch based reliability: r≥0.70 acceptable, r≥0.80 good, r≥0.90 excellent.

§ Cronbach’s alpha ≥0.70 acceptable.

Lastly, we evaluated scale-to sample targeting, item separation, person reliability and separation, and internal consistency reliability. Four scales had mean person ability values within ±1.0 logits of mean item difficulty. Mean person ability ranged from −6.27 to 3.98 logits (standard error range 0.43-2.06 logits). Supplementary figure S2 shows the item person maps for SBQ-LC’s scales. Item separation values ranged from 1.34 to 13.86 across the 17 scales. Person reliability ranged from 0.34 to 0.87 and person separation indices ranged from 0.71 to 2.56. Values for Cronbach’s alpha, as further evidence of internal consistency reliability, ranged from 0.56 to 0.91.

## Discussion

In this study we developed and validated SBQ-LC, a Rasch developed multi-domain item bank and modular instrument measuring symptom burden in people with long covid. SBQ-LC was developed in accordance with international, consensus based standards and regulatory guidance and can be used to evaluate the impact of interventions and to inform clinical care.^15^ ^23^ ^61^ We used the findings from published systematic reviews to construct a conceptual framework and generate an initial item pool. Rigorous content validity testing provided evidence of SBQ-LC’s relevance, comprehensiveness, comprehensibility, and acceptability. Rasch analysis guided optimisation of SBQ-LC’s items and response scales to construct an interval level instrument ready for psychometric evaluation using traditional indicators.

SBQ-LC was developed with the extensive involvement of adults with lived experience of long covid, and patient input is a strength of this study. Involvement of the target population is regarded as ideal in the development of instruments to measure patient reported outcomes and may be considered of particular importance in the context of long covid where the evidence base is rapidly evolving and affected individuals have reported experiences of stigma and a lack of acknowledgement from the medical community about the breadth and nature of their symptoms.^10^ Involvement of adults with long covid in all phases of the study (development, refinement, and validation) ensured patients’ voices were embodied in SBQ-LC’s items.

Rasch analysis of SBQ-LC guided reduction in the number of items and refinement of the rating scale, optimising the scales’ measurement accuracy and minimising respondent burden. Despite evidence of model fit, several of SBQ-LC’s scales were off-target, with low reliability values (measured by person separation and reliability and Cronbach’s alpha). Poor scale-to sample targeting is indicative of items within a scale failing to provide full coverage of person locations (ie, range of symptom burden experienced by the sample).^62^ Negative mean person measures (>1.0 logits), floor effects, and positively skewed distributions of response categories suggested SBQ-LC might be targeting individuals with higher levels of symptom burden than the level of burden represented by the field test sample. Highly skewed scoring distributions and poor targeting can produce low reliability coefficients even if an instrument is functioning as intended, providing a possible explanation for the low person reliability and alpha values observed for some of SBQ-LC’s scales.^62^ ^63^ In the first instance, a further Rasch analysis conducted in a representative clinical sample is required to confirm these findings. Scales remaining off-target will require critical review and further refinements (eg, creation of additional items to improve coverage of person locations) considered.

As a Rasch developed instrument, SBQ-LC’s ordinal raw scales may be converted to linear scales, with each 1 point change in a scale score being equidistant across the entire scale. Linear scores will enable the direct comparison of scores across SBQ-LC’s scales for a comprehensive assessment of symptom burden. As a multi-domain item bank, the modular construction of SBQ-LC means researchers and clinicians have the option of selecting only those scales required to provide targeted assessment of a particular symptom domain, thereby reducing respondent burden by removing the need to complete SBQ-LC in its entirety. Moreover, the Rasch model makes it possible to compare data from the SBQ-LC with other instruments measuring patient reported outcomes through co-calibration studies. As each item of a Rasch derived scale functions independently from others on that scale, SBQ-LC can be adapted to construct short forms, profile tools, or computer adaptive tests.^42^ A computer adaptive test is administered via a computer, which adapts to the respondent’s ability in real time by selecting different questions from an item bank to provide a more accurate measure of the respondent’s ability without the need to administer a large number of items.^64^ These tests can reduce respondent burden, improve accuracy, and provide individualised assessment—instrument characteristics that are attractive when assessing a health condition with heterogeneous, relapsing, and remitting symptoms such as long covid.

The burden of long covid on healthcare systems continues to grow as more people become infected with SARS-CoV-2.^65^ To meet this growing demand, services require cost effective resources to support safe, effective clinical management. The use of SBQ-LC in the TLC study will provide early evidence of SBQ-LC’s feasibility for use in remote patient monitoring. A previous randomised controlled trial has shown that remote symptom monitoring using patient reported outcomes can result in fewer attendances to emergency departments, reduce hospital admissions, prompt earlier intervention, and improve patients’ health related quality of life.^66^ If SBQ-LC is used in a clinical trial, symptom data collected remotely could provide valuable information on the safety, efficacy, and tolerability of new interventions for long covid.^16^ If used within routine care, SBQ-LC has potential to facilitate patient-clinician conversations, guide treatment decision making, and facilitate referrals to specialist services.^67^ ^68^ ^69^

### Limitations of this study

Sample representativeness is a limitation of this study. The personal characteristics of the content validation study sample were highly skewed and the use of social media for recruitment meant it was not possible to confirm the representativeness of the field test sample, including clinical evidence of covid-19 infection. The personal characteristics of the study sample (respondents were mostly female, of white ethnicity, older, with several comorbidities) were, however, consistent with large, UK based epidemiological studies reporting on the prevalence of long covid symptoms.^70^ ^71^ Findings from the REACT-2 (Real-time Assessment of Community Transmission 2) study, a cross sectional observational study of a community based sample, found that the persistence of one or more SARS-CoV-2 symptoms for 12 or more weeks was higher in women and increased linearly with age. Asian ethnicity was associated with lower risk of persistent symptoms compared with people of white ethnicity.^71^ The UK Office for National Statistics reported the prevalence of self-reported long covid to be highest in people aged 35 to 69 years, females, and those with another activity limiting health conditions or disabilities.^70^ A large retrospective cohort study on the incidence and co-occurrence of long covid features found white and non-white ethnicities to be affected equally.^11^ These studies suggest the field test sample in our study is broadly consistent with prevalence trends for long covid in the UK and that symptom reporting through SBQ-LC should not be substantially different for people of white versus non-white ethnicity. Nonetheless, further psychometric evaluation of SBQ-LC undertaken in a clinically confirmed, representative sample (with oversampling of underserved groups) remains a priority. Validation in patients not admitted to hospital will be undertaken as part of the TLC study, where potential participants will be identified from UK primary care practices to recruit a representative sample. Further work to validate SBQ-LC in a cohort of patients with long covid who were admitted to hospital with SARS-CoV-2 infection is planned.^24^

The relatively low response rate (37%), although within the typical range for electronic surveys, suggested potential field test participants (ie, possibly people experiencing higher levels of symptom burden) may have been deterred by the consenting and onboarding process or lacked sufficient incentive to participate.^72^ Personal information was not collected for people who opted not to participate, precluding analysis of the personal characteristics of non-respondents. The high completion rate (83%) suggested that most participants, once onboarded to Atom5, were able to complete the full SBQ-LC.

Validation of SBQ-LC is planned as part of the TLC study to confirm the study findings. Further Rasch analysis and an evaluation of SBQ-LC using traditional psychometric indicators (test-retest reliability, construct validity, responsiveness, and measurement error) will be undertaken. Studies to explore the feasibility and acceptability of SBQ-LC for use in health and social care settings are also needed and will help to inform guidance on the use of SBQ-LC in routine care. SBQ-LC is currently available in UK English as an electronic patient reported outcome and in paper form. Linguistic and cross cultural validation studies will ensure SBQ-LC is suitable for use in a range of health and social care settings in the UK and in other countries, including low and middle income countries.^73^

### Conclusions

The presence of symptoms of covid-19 persisting beyond the acute phase of infection in a considerable number of patients represents an ongoing challenge for healthcare systems globally. High quality instruments to measure patient reported outcomes are required to better understand the signs, symptoms, and underlying pathophysiology of long covid, to develop safe and effective interventions, and to meet the day-to-day needs of this growing patient group. SBQ-LC was developed as a comprehensive measure of the symptom burden from long covid. With promising psychometric properties, SBQ-LC is available for use in long covid research studies and in the delivery of clinical care.

**What is already known on this topic**

As of December 2021, 1.3 million people in the United Kingdom and an estimated >100 million worldwide are currently living with long covid or post-covid-19 syndrome; this figure will continue to rise as more people are affected with SARS-CoV-2 infection
Studies have shown that long covid is a novel, multisystem condition with considerable symptom burden and negative impacts on work capability and quality of life
Owing to a lack of patient reported outcome measures specific to long covid, researchers and clinicians are using bespoke surveys, generic patient reported outcome measures, or symptom burden measures validated in other disease groups to assess the symptom burden from long covid

**What this study adds**

With extensive patient involvement, this mixed methods study developed and validated the symptom burden questionnaire for long covid
This novel questionnaire has the potential to benefit international clinical trials and inform best practice in clinical management

## Data Availability

Data for this project are not currently available for access outside the Therapies for Long COVID Study research team. The dataset may be shared when finalised, but this will require an application to the data controllers. The data may then be released to a specific research team for a specific project dependent on the independent approvals being in place.

## Web extra

Extra material supplied by authors

## Notes

Contributors: MJC, SH, SEH, and OLA developed the concept and design of the SBQ-LC. EHD and CF conceptualised and designed delivery of the SBQ-LC in Atom5. MJC, SH, SEH, AS, CM, OLA, GMT, LJ, EHD, and GP obtained funding. SH and MJC supervised the study. SEH, SH, OLA, GMT, CM, and MJC developed the study design and methodology. MJC, SH, and SEH were responsible for project management. AW and GM provided administrative and project management support. OLA, GP, KM, JO, JC, SEH, CM, and CF were responsible for data acquisition. SEH, AS, LJ, and CF were responsible for data curation and validation. SEH, SH, LJ, and MJC did the analyses. SEH wrote the original manuscript. SEH, SH, and MC are the guarantors. All authors provided critical revisions and approved the final manuscript. The corresponding author attests that all authors meet authorship criteria and that no others meeting the criteria have been omitted.

The manuscript’s guarantors (SEH, SH, and MC) affirm that the manuscript is an honest, accurate, and transparent account of the study being reported; that no important aspects of the study have been omitted; and that any discrepancies from the study as originally planned have been explained.

Dissemination to participants and related patient and public communities: We will distribute the article to clinicians and long covid support groups. We will distribute findings on social media, and a plain language summary on the Therapies for Long COVID Study website (www.birmingham.ac.uk/research/applied-health/research/long-covid/index.aspx). We will share findings through conference presentations, including invited talks and webinars. Information on the symptom burden questionnaire for long covid (SBQ-LC) and obtaining a license for use is available at www.birmingham.ac.uk/sbq.

Provenance and peer review: Not commissioned; externally peer reviewed.
