# Supplementary material for: Development and validation of the symptom burden questionnaire for long covid (SBQ-LC): Rasch analysis
Source: BMJ. 2022 Apr 27;377:e070230. doi: 10.1136/bmj-2022-070230 (PMC9043395; doi:10.1136/bmj-2022-070230)

Development and validation of the Symptom Burden Questionnaire for Long COVID: a Rasch analysis

Description of Supplementary Material

Table S1. Concept coverage matrix mapping symptom coverage of the candidate PROs to the SBQ-LC's conceptual framework

Table S2. Item-level content validity indices (I-CVI) per item for the draft SBQ-LC item pool based on the clinician survey of item/symptom relevance.

Table S3. Themes and exemplar quotations from content validation of the SBQ-LC item pool: cognitive debriefing (n = 13) and clinician survey (n =10).

Table S4. Symptom coverage of the SBQ-LC (Version 1.0) per domain of the conceptual framework.

File S1. Clinician survey for content validation of the draft SBQ-LC item pool.

File S2. Exemplar SBQ-LC "Breathing" scale illustrating the respondent instructions, item wording and response scales

Figure S1. Category probability curves and category-level fit statistics showing rating scale function per item of the final SBQ-LC (Version 1.0)

For each graph, the y-axis represents the expected probability of endorsement of any given category when a person responds to the item. The x-axis represents the person ability relative to the item difficulty, with origins set to 0. The scale is measured in logits. Locations to the right of '0' represent greater and greater levels of symptom burden. Moving to the left of '0' locations represent lower levels of symptom burden. Categories should advance in ascending order from left to right along the x-axis and each have a distinct peak (indicating that the category is the most probable (modal) category at that point on the latent variable). The crossover points between two curves are the equal probability points of thresholds. Category measures and category thresholds presented in the corresponding table should advance montonically. Category outfit MnSQ values > 2.0 logits are indicative of model fit.

Figure S2. Item-person maps for the 17 scales of the final SBQ-LC (Version 1.0)
For each scale, the person-item map displays the location of person abilities and item difficulties respectively along the same latent dimension (y-axis).

Table S1. Concept coverage matrix mapping symptom coverage of the candidate PROs to the SBQ-LC's conceptual framework

Symptoms reported in Aiyegbusi et al. 2021[1]		PHOSP-COVID Patient Symptom Questionnaire[2]	C-19 YRS[3]	Rotterdam Symptom Checklist[4]	MD Anderson Symptom Inventory - Core Items[5]	Flu-PRO (Items mapped from Flu-PRO conceptual framework)[6]	PRO-CTCAE[7]
Cardiopulmonary	fatigue	X	X		X		X
	shortness of breath (dyspnoea)	X	X	X	X	X	X
	shortness of breath at rest	X	X				
	shortness of breath upon exertion	X	X				
	chest tightness	X				X	
	chest pain	X	X				
	palpitations	X					X
	wheezing		X				X
Nasopharyngeal	loss of smell (anosmia)	X					
	rhinitis/rhinorrhoea					X	
	sneezing					X	
	chronic sinusitis/congestion					X	
	dysgeusia (altered taste)	X					X
	sore throat		X			X	
	cough	X	X			X	X
	sputum production					X	
	hoarse voice/voice change	X	X				X
	aphonia						
	ear pain						
	hearing loss						
	tinnitus	X					X
Gastroenterological	abdominal pain	X	X	X		X	X
	nausea	X		X	X	X	X
	vomiting	X		X	X	X	X
	diarrhoea	X	X	X		X	X
	loss of appetite	X			X	X	X
	gastritis (indigestion)			X			
	weight loss/anorexia	X	X				

Symptoms reported in Aiyegbusi et al. 2021[1]		PHOSP-COVID Patient Symptom Questionnaire[2]	C-19 YRS[3]	Rotterdam Symptom Checklist[4]	MD Anderson Symptom Inventory - Core Items[5]	Flu-PRO (Items mapped from Flu-PRO conceptual framework)[6]	PRO-CTCAE[7]
MSK	joint pain	X	X				X
	muscle pain (myalgia)	X	X	X			X
Neuropsychological	memory loss (amnesia)	X	X		X		X
	difficulty thinking	X	X				
	brain fog	X					
	inability to concentrate	X	X	X			X
	disorientation						
	delirium						
	sleep disorder (e.g., insomnia)	X	X	X	X		X
	moderate-to-severe depression		X				
	moderate-to-severe anxiety		X	X			
	mood change	X		X			X
	self-harm/suicide		X				
	neuralgia/ neuropathy						
	needle pains arms & legs	X		X	X		X
	tremors	X					
	seizures	X					
	visual disturbances	X					X
Cutaneous	skin changes						
	hair loss			X			X
	itching						X
	skin rash	X	X				X
	spots of feet	X					
Miscellaneous	fever	X	X		X		
	chills			X		X	X
	sweats					X	X
	hot flushes						X
	headache	X	X	X		X	X
	sicca syndrome						
	red eye/eye irritation			X		X	
	dizziness/vertigo	X		X			X

Symptoms reported in Aiyegbusi et al. 2021[1]		PHOSP-COVID Patient Symptom Questionnaire[2]	C-19 YRS[3]	Rotterdam Symptom Checklist[4]	MD Anderson Symptom Inventory - Core Items[5]	Flu-PRO (Items mapped from Flu-PRO conceptual framework)[6]	PRO-CTCAE[7]
	asthenia/weakness	X	X			X	
	unspecified pain/body ache	X	X		X	X	X
	bladder incontinence	X	X				X
	ulcer						
	**Total No Symptoms**	**38**	**27**	**17**	**10**	**19**	**31**
	**Total Coverage (%)**	**60.3**	**42.9**	**27.0**	**15.9**	**30.2**	**49.2**

References:

1 Aiyegbusi OL, Hughes SE, Turner G, *et al.* Symptoms, complications and management of long COVID: a review. *J R Soc Med* 2021;114:428–42. doi:10.1177/01410768211032850

2 Evans RA, McAuley H, Harrison EM, *et al.* Physical, cognitive, and mental health impacts of COVID-19 after hospitalisation (PHOSP-COVID): a UK multicentre, prospective cohort study. *The Lancet Resp Med* 2021;9:1275–87. doi:10.1016/S2213-2600(21)00383-0

3 O'Connor RJ, Preston N, Parkin A, *et al.* The COVID-19 Yorkshire Rehabilitation Scale (C19-YRS): application and psychometric analysis in a post-COVID-19 syndrome cohort. *J Med Virol*. doi:10.1002/jmv.27415

4 de Haes J, van Knippenberg FCE, Neijt JP. Measuring psychological and physical distress in cancer patients: structure and application of the Rotterdam Symptom Checklist. *Br J Cancer* 1990;62:1034–8. doi:10.1038/bjc.1990.434

5 Cleeland CS, Mendoza TR, Wang XS, *et al.* Assessing symptom distress in cancer patients: the M.D. Anderson Symptom Inventory. *Cancer* 2000;89:1634–46. doi:10.1002/1097-0142(20001001)89:7<1634::aid-cncr29>3.0.co;2-v

6 Powers JH, Guerrero ML, Leidy NK, *et al.* Development of the Flu-PRO: a patient-reported outcome (PRO) instrument to evaluate symptoms of influenza. *BMC Infect Dis* 2016;16:1. doi:10.1186/s12879-015-1330-0

7 Basch E, Reeve BB, Mitchell SA, *et al.* Development of the National Cancer Institute's Patient-Reported Outcomes Version of the Common Terminology Criteria for Adverse Events (PRO-CTCAE). *J Natl Cancer Inst* 2014;106:dju244. doi:10.1093/jnci/dju244

Table S2. Item-level content validity indices (I-CVI) per item for the draft SBQ-LC item pool based on the clinician survey of item/symptom relevance (n = 10)

SBQ-LC v0.1.0 item (symptom)	I-CVI[a]/ κ^b
shortness of breath (difficulty breathing)	1.0
shortness of breath (difficulty breathing) when at rest	1.0
shortness of breath (difficulty breathing) when climbing a flight of stairs	1.0
chest tightness	1.0
chest pain	1.0
wheezing (noisy breathing)	1.0
shortness of breath (difficulty breathing) when lying flat	1.0
wake up at night short of breath	1.0
fatigue	1.0
low energy levels	1.0
tiredness	1.0
problems falling asleep	1.0
sleep short and interrupted	1.0
brain fog	1.0
difficulty concentrating	1.0
problems concentrating	1.0
feel confused	1.0
tingling and numbness in arms and legs	1.0
balance problem	1.0
difficulty planning	1.0
problems focussing on a single task	1.0
lack of interest in things around you	1.0
feelings of being depressed	1.0
anxiety	1.0
feel stressed	1.0
feel lonely	1.0
loss of smell	1.0
cough	1.0
loss of taste	1.0
loss of appetite	1.0
muscle pain	1.0
joint pain	1.0
muscle weakness	1.0
headache (severity)	1.0
headache (frequency)	1.0
fever (severity)	1.0
fever (frequency)	1.0
ability to carry out your usual activities (work, school, shopping, housework)	1.0
interfere with relationships with others (friends and family)	1.0
ability to carry out your usual activities (work, school, shopping, housework)	1.0
ability to get around easily	1.0
interfere with mood	1.0
interfere with quality of life	1.0
palpitations (a pounding, fluttering or irregular heartbeat)	0.9
rapid breathing (panting)	0.9
swelling of your legs and/or feet	0.9
problems with thinking and planning	0.9
problems remembering	0.9
disorientation	0.9
self-harm	0.9
feel like the person you were before illness	0.9
mood swings	0.9
production of mucus/phlegm	0.9
hoarse voice	0.9

SBQ-LC v0.1.0 item (symptom)	I-CVI[a]/ κ^b
problem swallowing food or drink	0.9
abdominal pain	0.9
nausea	0.9
weight loss	0.9
diarrhoea	0.9
joint swelling	0.9
joint stiffness	0.9
rash	0.9
chills/shivering	0.9
problem with sweating	0.9
aching all over the body	0.9
pain on breathing	0.8
feel faint or have blackouts	0.8
memory loss	0.8
muscle twitching or cramping	0.8
word-finding difficulty	0.8
problem understanding others	0.8
sinus congestion	0.8
stuffy or runny nose	0.8
sore throat	0.8
changes to your voice	0.8
new hearing loss	0.8
vomiting	0.8
indigestion	0.8
abdominal bloating	0.8
bruising (that happened for no obvious reason)	0.8
red eyes	0.8
dry eyes	0.8
changes to your vision	0.8
blurred vision	0.8
flashing lights	0.8
vertigo (dizziness)	0.8
swelling of your hands and/or arms	0.7
cold hands/feet	0.7
shooting or stabbing pain	0.7
aching or burning pain	0.7
tremors (uncontrollable shaking or trembling in part of your body)	0.7
seizures (fits)?	0.7
movement and coordination difficulty	0.7
earache (ear pain)	0.7
changes to your hearing	0.7
choke when eating or drinking	0.7
constipation	0.7
skip meals	0.7
loss of bowel control	0.7
itchy skin	0.7
hives (raised, itchy patches)	0.7
sensitivity to light	0.7
excessive tears (watery eyes)	0.7
hot flushes	0.7
sneezing	0.60
tinnitus (noises or ringing sounds in your ears)	0.60
dry mouth	0.60
sensitivity of throat	0.60
inflamed nose	0.60
dry/scaly skin	0.60
purple-red spots	0.60

SBQ-LC v0.1.0 item (symptom)	I-CVI[a]/ κ[b]
itchy eyes	0.60
pressure behind the eyes	0.60
floaters (shapes people can see drifting across their vision)	0.60
loss of control of urine	0.60
hallucinations	0.50
slurred speech	0.50
cough up blood	0.50
weight gain	0.50
hair loss	0.50
swelling of lips/face	0.50
avoid certain food or drinks	0.40
ulcer	0.40

[a]I-CVI, item-level content validity index for item relevance.

[b]kappa designating agreement on relevance: k* (I-CVI - pc)/(1.0-pc) described in Poilit et al. (2007).

[c]Evaluation criteria for kappa, using guidelines proposed by Cicchetti and Sparrow (1981) and described in Polit et al. (2007): Fair = κ = 0.40 -0.59; Good = κ = 0.60 -0.74; Excellent = κ > 0.74

Table S3. Themes and exemplar quotations from content validation of the SBQ-LC item pool: cognitive debriefing (n = 13) and clinician survey (n = 10).

Theme	Exemplar quote
Content relevance	*"...The only the only question that I sort of went...... was number seven around how often did you feel like you were like the person you were before your illness. And I think that's probably the most important [question] .. yeah, that was the most emotive one for me...."* – Participant 02
Content comprehensiveness	*"...the questions are unique in the way they capture, show understanding of, and validate the range of symptoms a person with Long COVID can experience..."* – Participant 12<br><br>*I think you really need a section for neuro, neurological aspects in there. And the eyes, bloodshot eyes, and things like that but otherwise I think you, you kind of captured most of the symptoms, if I think.* -Participant 10<br><br>*"No, I think I think it is really good that it's structured this way and it's been detailed in terms of the shortness at the breath, fatigue, the muscle and joint pain. Because even me, I couldn't describe although I feel them, I don't have a medical knowledge. Expanding and going into details, I think it's really good, I think it is really helpful for whoever is going to take the questionnaire. It's very helpful."* – Participant 13
Item clarity	*"...'Fatigue', to me, is where I feel like I need to sleep. I'm probably sleeping between 10 and 11 hours and the moment and then napping in the day. I think 'low energy' is kind of having that draining feeling."* – Participant 01
Item acceptability	*"[I] thought it was relatively straightforward, I would say. My concentration isn't good, and I managed."* - Participant 12
Response scales	*"...the difficulty is around the fluctuation and the seven-day window. For example, breathlessness is a big one for me. I can go weeks and feel ok, and then, all of a sudden, it relapses and I'm struggling to breathe again"* - Participant 02

Table S4. Symptom coverage of the SBQ-LC (Version 1.0) per domain of the conceptual framework (n = 17)

**Breathing**
Shortness of breath (sitting)
Shortness of breath (climbing stairs)
Shortness of breath (lying flat)
Wake up short of breath
Breathing faster than usual
Chest tightness
Wheezing (noisy breathing)

**Pain**
Chest pain
Pain on breathing
Shooting or stabbing pain
Aching or burning pain

**Circulation**
Palpitations (heart skips a beat/pounding heartbeat)
Feeling faint (lightheaded)
Dizziness on standing
Swelling of legs/feet
Hands/feet colder than usual

**Fatigue**
Fatigue (physical/mental exhaustion)
Low energy
Tiredness (need for sleep)
Worsening symptoms after activity

**Memory, Thinking & Communication**
Difficulty remembering
Memory loss
Brain fog
Confusion/Knowing what is happening
Difficulty concentrating
Difficulty planning
Word finding difficulty
Difficulty understanding what others were saying
Slurred speech
Reading difficulty (not dyslexia)

**Movement**
Tremor (uncontrollable shaking or trembling)
Balance difficulty
Movement and coordination difficulty

**Ear, Nose & Throat**
Altered smell (foods/objects smelling different to usual)
Altered taste (foods tasting different to usual)
Sneezing
Stuffy, runny nose
Sinus congestion
Production of mucus (phlegm)
Cough
Sore Throat
Hoarse voice (change in voice quality)
Difficulty swallowing food or drink
Ear pain
New hearing loss
Tinnitus (Noises or ringing sounds in your ears)
Sensitivity to sound

**Stomach & Digestion**
Belly/tummy pain
Bloating
Nausea (urge to vomit)
Indigestion and heartburn
Weight loss
Weight gain
Diarrhoea
Constipation

**Muscles & Joints**
Muscle pain
Muscle weakness
Muscle stiffness
Joint pain
Joint swelling
Joint stiffness
Muscle twitching
Muscle cramping
Tingling and numbness (pins and needles)

**Mental Health & Wellbeing**
Lack of interest
Feeling anxious
Feeling sad
Thoughts about self-harm
Mood swings
Change in appetite
Feeling lonely or unsupported
Feeling hopeful
Loss of identity

Skin & Hair
Dry skin
Scaly skin
Itchy skin
Purple-red spots on feet
Rash
Hives (welts or raised itchy patches of skin)
Hair loss
Nail changes (ridging, pitting, discolouration or brittle nails)

Female Reproductive & Sexual Health
Changes to menstrual period
Worsening premenstrual syndrome (PMS)
Passing blood clots during period
Vaginal dryness
Vaginal discharge
Difficulty with orgasm/climax
Decreased interest in sex

Eyes
Red or bloodshot eyes
Dry eyes
Itchy eyes
Blurred and/or double vision
Flashing lights and floaters (small dark shapes that float across your vision)
Sensitivity to light
Watery eyes (excessive tears)
Pressure behind the eyes
Pain behind the eyes
Discomfort when blinking

Female Reproductive & Sexual Health
Changes to menstrual period
Worsening premenstrual syndrome (PMS)
Passing blood clots during period
Vaginal dryness
Vaginal discharge
Difficulty with orgasm/climax
Decreased interest in sex

Male Reproductive & Sexual Health
Difficulty getting/keeping an erection
Difficulty with ejaculation
Decreased interest in sex

Other symptoms
Fever
Chills and shivering
Sweating
Hot flushes
Aching all over the body
Swelling of glands (lymph nodes)
Vertigo (feeling like everything spinning around you)
Swelling of face, lips, tongue and/or throat
Reaction to known allergies
Reaction to new allergies
Loss of control of urine
Difficulty passing urine
Passing more urine than usual
Increased thirst
Mouth ulcers
Worsening of known dental problems
Dry mouth
Headache

Impact on Daily Life
Work, education & organised activities
Shopping
Around the house
Ability to move around
Self-care
Relationships
Socialising
Enjoyment of life

THE TLC STUDY | Therapies for Long COVID in non-hospitalised individuals

Content validation of the Symptom Burden Questionnaire™ for Long COVID (SBQ™-LC): Professionals' perspectives

1. Welcome and Introduction to the Survey

As part of the Therapies for Long COVID (TLC) Study, we are developing a new patient-reported outcome measure (PROM) to measure symptom burden in individuals with Long COVID.

This new PROM is called the **Symptom Burden Questionnaire™ for Long COVID** or SBQ™-LC. As part of the development and validation process we need to test the draft questions with professionals and patients to make sure the questions are relevant, provide comprehensive coverage of the symptoms of Long COVID, and are acceptable and understandable.

By completing this survey, you are helping us to test the draft questions by giving us your views as a health/medical professional. The findings will be used to refine the questions, develop clinical alerting thresholds, and construct the SBQ™-LC.

The survey should take approximately 30 minutes to complete. There is a free text box at the end of each section and we welcome suggested edits/rewording. Please note that the question number is for development purposes only and will not be visible to patients in the final questionnaire. The ordering of questions in this survey does not reflect any form of prioritisation and is not how the questions will be ordered in the final version of the SBQ™-LC.

As the SBQ™-LC is currently under development, please treat this survey as confidential and do not use or share any of its content.

If you have any questions about the SBQ™-LC, please contact Sarah Hughes, Research Fellow, Centre for Patient Reported Outcome Research (CPROR), University of Birmingham at s.e.hughes@bham.ac.uk.

Thank you for giving up your time to help with the TLC project.

2. Symptom Domain: Cardiopulmonary

1. Please rate each of the following items for its relevance as a symptom of Long COVID and its clarity (ease of understanding from a patient perspective): *

	Relevance	Clarity
CP01 In the last 7 days, how severe was your **shortness of breath** (difficulty breathing) at its worst?		
CP02 In the last 7 days, how severe was your **shortness of breath (difficulty breathing) when at rest** at its worst?		
CP03 In the last 7 days, how **severe was your shortness of breath (difficulty breathing) when climbing a flight of stairs** at its worst?		
CP04 In the last 7 days, how severe was the **tightness of your chest** at its worst?		
CP05 In the last 7 days, how severe was **your chest pain** at its worst?		
CP06 In the last 7 days, how severe were your **palpitations** (a pounding, fluttering or irregular heartbeat) at its worst?		
CP07 In the last 7 days, how severe was your **wheezing (noisy breathing)** at its worst?		
CP08 In the last 7 days, how severe was **your pain on breathing** at its worst?		
CP10 In the last 7 days, how often did you **feel faint or have blackouts**?		
CP11 In the last 7 days, how severe was the **swelling of your hands and/or arms** at its worst?		
CP12 In the last 7 days, how severe was y**our shortness of breath (difficulty breathing) when lying flat** at its worst?		
CP13 In the last 7 days, how often did you **wake up at night short of breath**?		
CP14 In the last 7 days, how often did you have		

	Relevance	Clarity
**rapid breathing (panting)**?		
CP15 In the last 7 days, how often did you have **cold hands/feet**?		
CP16 In the last 7 days, how severe was the **swelling of your legs and/or feet** at its worst?		

Please add any further information to help us improve these questions. Please include the question ID number along with any edits or other proposed changes.

3. Symptom Domain: Fatigue

2. Please rate each of the following items for its relevance as a symptom of Long COVID and its clarity (ease of understanding from a patient perspective): *

	Relevance	Clarity
F01 In the last 7 days, how severe was **your fatigue** at its worst?		
F02 In the last 7 days, how often did you have **low energy levels**?		
F03 In the last 7 days, how severe was your **tiredness** at its worst?		

Please add any further information to help us improve these questions. Please include the question ID number along with any edits or other proposed changes.

4. Symptom Domain: Neurological 1

3. Please rate each of the following items for its relevance as a symptom of Long COVID and its clarity (ease of understanding from a patient perspective): *

	Relevance	Clarity
N01 In the last 7 days, how often did you have **problems falling asleep**?		
N02 In the last 7 days, how often was your **sleep short and interupted**?		
N03 In the last 7 days, how severe were your **problems with thinking and planning** at their worst?		
N04 In the last 7 days, how often did you have **problems remembering things**?		
N05 In the last 7 days, how severe was your **memory loss** at its worst?		
N06 In the last 7 days, how severe was your **brain fog** at its worst?		
N07 In the last 7 days, how severe was your **difficulty concentrating** at its worst?		
N08 In the last 7 days, how often did you have **problems concentrating**?		
N09 In the last 7 days, how often did you **feel confused about where you were, where you were going or what was happening**?		
N10 In the last 7 days, how severe was your **disorientation** at its worst?		
N12 In the last 7 days, how severe was your **shooting or stabbing pain** at its worst?		
N13 In the last 7 days, how severe was your **aching or burning pain** at its worst		

Please add any further information to help us improve these questions. Please include the question ID number along with any edits or other proposed changes.

** Neurological questions are continued on the next page.

5. Symptom Domain: Neurological 2

4. Please rate each of the following items for its relevance as a symptom of Long COVID and its clarity (ease of understanding from a patient perspective): *

	Relevance	Clarity
N14 In the last 7 days, how severe was your **muscle twitching or cramping** at its worst?		
N15 In the last 7 days, how severe was the **tingling and numbness in your arms and legs** at its worst?		
N16 In the last 7 days, how severe were your **tremors (uncontrollable shaking or trembling in part of your body)** at its worst?		
N17 In the last 7 days, how often did you have **seizures (fits)**?		
N19 In the last 7 days, how severe was your **balance problem** at its worst?		
N20 In the last 7 days, how severe was your problem with **movement and coordination** at its worst?		
N21 In the last 7 days, how severe was your **word-finding difficulty** at its worst?		
N22 In the last 7 days, how severe was your **problem understanding others** at its worst?		
N23 In the last 7 days, how severe was your **difficulty planning** at its worst?		
N24 In the last 7 days, how often did you have **problems focussing on a single task**?		
N25 In the last 7 days, how often did you have **hallucinations**?		
N26 In the last 7 days, how severe was your **slurred speech** at its worst?		

Please add any further information to help us improve these questions. Please include the question ID number along with any edits or other proposed changes.

6. Symptom Domain: Psychological

5. Please rate each of the following items for its relevance as a symptom of Long COVID and its clarity (ease of understanding from a patient perspective): *

	Relevance	Clarity
PSY01 In the last 7 days, how often did you feel a **lack of interest in things around you**?		
PSY02 In the last 7 days, how severe were your feelings of **being depressed** at their worst?		
PSY03 In the last 7 days, how severe was your **anxiety** at its worst?		
PSY04 In the last 7 days, did you have **thoughts about harming yourself** in anyway?		
PSY07 In the last 7 days, how often did you **feel like the person you were before your illness**?		
PSY08 In the last 7 days, how severe were your **mood swings** at their worst?		
PSY09 In the last 7 days, how often did you **feel stressed**?		
PSY10 In the last 7 days, how often did you **feel lonely**?		

Please add any further information to help us improve these questions. Please include the question ID number along with any edits or other proposed changes.

7. Symptom Domain: Ear, Nose, and Throat 1

6. Please rate each of the following items for their relevance as a symptom of Long COVID and their clarity (ease of understanding from a patient perspective): *

	Relevance	Clarity
ENT01 In the last 7 days, how severe was your **sinus congestion** at its worst?		
ENT02 In the last 7 days, how severe was your **loss of smell** at its worst?		
ENT03 In the last 7 days, how severe was your **stuffy or runny nose** at its worst?		
ENT04 In the last 7 days, how severe was your **sneezing** at its worst?		
ENT05 In the last 7 days, how severe was your **cough** at its worst?		
ENT06 In the last 7 days, how severe was your **loss of taste** at its worst?		
ENT07 In the last 7 days, how severe was your **sore throat** at its worst?		
ENT08 In the last 7 days, how severe was your production of **mucus/phlegm** at its worst?		
ENT09 In the last 7 days, how severe was your **hoarse voice** at its worst?		
ENT10 In the last 7 days, how severe were the **changes to your voice** at their worst?		
ENT11 In the last 7 days, how often did you **cough up blood**?		

Please add any further information to help us improve these questions. Please include the question ID number along with any edits or other proposed changes.

**** Ear, Nose and Throat questions are continued on the next page.**

8. Symptom Domain: Ear, Nose, and Throat 2

7. Please rate each of the following items for their relevance as a symptom of Long COVID and their clarity (ease of understanding from a patient perspective): *

	Relevance	Clarity
ENT12 In the last 7 days, how severe was your **earache (ear pain)** at its worst?		
ENT13 In the last 7 days, how severe were the **changes to your hearing**?		
ENT14 In the last 7 days, how severe was your **new hearing loss** at its worst?		
ENT15 In the last 7 days, how severe was your **tinnitus (noises or ringing sounds in your ears)** at its worst?		
ENT16 In the last 7 days, how severe was your **dry mouth** at its worst?		
ENT17 In the last 7 days, how severe was the **sensitivity of your throat**?		
ENT18 In the last 7 days, how often did you **avoid certain food or drinks**?		
ENT19 In the last 7 days, how often did you **choke when eating or drinking**?		
ENT20 In the last 7 days, how severe was your **problem swallowing food or drink**?		
ENT21 In the last 7 days, how severe was your **inflammed nose** at its worst?		

Please add any further information to help us improve these questions. Please include the question ID number along with any edits or other proposed changes.

9. Symptom Domain: Gastroenterological

8. Please rate each of the following items for their relevance as a symptom of Long COVID and their clarity (ease of understanding from a patient perspective): *

	Relevance	Clarity
GI01 In the last 7 days, how severe was your **abdominal pain** at its worst?		
GI02 In the last 7 days, how severe was your **nausea** at its worst?		
GI03 In the last 7 days, how severe was your **vomiting** at its worst?		
GI04 In the last 7 days, how severe was your **indigestion** at its worst?		
GI05 In the last 7 days, how severe was your **loss of appetite** at its worst?		
GI06 In the last 7 days, how severe has your **weight loss** been?		
GI07 In the last 7 days, how severe was your **diarrhoea** at its worst?		
GI08 In the last 7 days, how severe was your **constipation** at its worst?		
GI09 In the last 7 days, how severe was your **abdominal bloating** at its worst?		
GI10 In the last 7 days, how often did you **skip a meal**?		
GI11 In the last 7 days, how severe was your **ulcer** at its worst		
GI12 In the last 7 days, how severe was your **loss of bowel control** at its worst?		
GI13 In the last 7 days, how severe was your **weight gain** at its worst?		

Please add any further information to help us improve these questions. Please include the question ID number along with any edits or other proposed changes.

10. Symptom Domain: Musculoskeletal

9. Please rate each of the following items for its relevance as a symptom of Long COVID and its clarity (ease of understanding from a patient perspective): *

	Relevance	Clarity
MSK01 In the last 7 days, how severe was your **muscle pain** at its worst?		
MSK02 In the last 7 days, how severe was your **joint pain** at its worst?		
MSK03 In the last 7 days, how severe was your **joint swelling** at its worst?		
MSK04 In the last 7 days, how severe was your **joint stiffness** at its worst?		
MSK05 In the last 7 days, how severe was your **muscle weakness** at its worst?		

Please add any further information to help us improve these questions. Please include the question ID number along with any edits or other proposed changes.

11. Symptom Domain: Cutaneous

10. Please rate each of the following items for its relevance as a symptom of Long COVID and its clarity (ease of understanding from a patient perspective): *

	Relevance	Clarity
CUT01 In the last 7 days, how severe was your **dry/scaly skin** at its worst?		
CUT02 In the last 7 days, how severe was your **hair loss**?		
CUT03 In the last 7 days, how severe was your **itchy skin** at its worst?		
CUT04 In the last 7 days, did you have **purple-red spots on your body**?		
CUT05 In the last 7 days, did you have a **rash**?		
CUT07 In the last 7 days, did you have **hives (raised, itchy patches)**?		
CUT08 In the last 7 days, how severe was your **bruising (that happened for no obvious reason)** at its worst?		

Please add any further information to help us improve these questions. Please include the question ID number along with any edits or other proposed changes.

12. Symptom Domain: Vision

11. Please rate each of the following items for its relevance as a symptom of Long COVID and its clarity (ease of understanding from a patient perspective): *

	Relevance	Clarity
VIS01 In the last 7 days, how often did you have **red eyes**?		
VIS02 In the last 7 days, how often did you have **dry eyes**?		
VIS03 In the last 7 days, how often did you have **itchy eyes**?		
VIS04 In the last 7 days, how severe were the **changes to your vision** at their worst?		
VIS05 In the last 7 days, how severe was your **blurred vision** at its worst?		
VIS06 In the last 7 days, how often did you have **flashing light in the eyes**?		
VIS07 In the last 7 days, how severe was the **feeling of pressure behind your eyes** at its worst?		
VIS08 In the last 7 days, how severe was your **sensitivity to light** at its worst?		
VIS09 In the last 7 days, how often did you have **floaters (shapes people can see drifting across their vision)**?		
VIS10 In last 7 days, how often did you have **excessive tears (watery eyes)**?		

Please add any further information to help us improve these questions. Please include the question ID number along with any edits or other proposed changes.

13. Symptom Domain: Systemic + Miscellaneous

12. Please rate each of the following items for its relevance as a symptom of Long COVID and its clarity (ease of understanding from a patient perspective). *

	Relevance	Clarity
SYS01 In the last 7 days, how severe was your **headache** at its worst?		
SYS02 In the last 7 days, how often did you have **headache**?		
SYS03 In the last 7 days, how severe was your **fever** at its worst?		
SYS04 In the last 7 days, how often did you **feel hot or feverish**?		
SYS05 In the last 7 days, how often did you have **chills/shivering**?		
SYS06 In the last 7 days, how severe was your problem with **sweating** at its worst?		
SYS07 In the last 7 days, how severe were your **hot flushes** at its worst?		
SYS08 In the last 7 days, how severe was your **vertigo (dizziness)** at its worst?		
SYS09 In the last 7 days, how severe was your **aching all over the body** at its worst?		
SYS10 In the last 7 days, how often did you have **loss of control of urine (leakage)**?		
SYS11 In the last 7 days, how severe was the **swelling of your face/lips** at its worst?		

Please add any further information to help us improve these questions. Please include the question ID number along with any edits or other proposed changes.

14. Interference

13. Please rate each of the following items for its relevance and its clarity (ease of understanding from a patient perspective). *

	Relevance	Clarity
INT01 In the last 7 days, have your symptoms interfered with your **ability to carry out your usual activities**? (work, school, shopping, housework)		
INT02 In the last 7 days, have your symptoms interfered with your **relationships with others (friends and family)**?		
INT03 In the last 7 days, have your symptoms interfered with your **ability to look after yourself (bathing and dressing)**?		
INT04 In the last 7 days, have your symptoms interfered with your **ability to get around easily**?		
INT05 In the last 7 days, have your symptoms interfered with your **mood**?		
INT06 In the last 7 days, have your symptoms interfered with your **quality of life**?		

Please add any further information to help us improve these questions. Please include the question ID number along with any edits or other proposed changes.

15. Clinical Alerts and Reporting Thresholds

14. Please identify any symptom that, in your professional opinion, would require an alert to be sent to the patient's clinical team. For each symptom, please indicate the level of symptom severity that you think should trigger an alert to the clinical team. *

	No alert	Alert if symptom is mild	Alert if moderate	Alert if severe
Shortness of breath (dyspnea)	☐	☐	☐	☐
Shortness of breath at rest	☐	☐	☐	☐
Shortness of breath upon exertion	☐	☐	☐	☐
Shortness of breath flat (orthopnea)	☐	☐	☐	☐
Shortness of breath - night (paroxysmal noctural dyspnoea)	☐	☐	☐	☐
Chest tightness	☐	☐	☐	☐
Chest pain	☐	☐	☐	☐
Palpitations	☐	☐	☐	☐
Wheezing	☐	☐	☐	☐
Fainting/blackouts	☐	☐	☐	☐
Limb swelling	☐	☐	☐	☐
Post-activity polypnea	☐	☐	☐	☐
Pain on breathing	☐	☐	☐	☐
Loss of smell (anosmia)	☐	☐	☐	☐
Rhinitis/rhinorhea	☐	☐	☐	☐
Sneezing	☐	☐	☐	☐
Chronic sinusitis/congestion	☐	☐	☐	☐
Inflammed nose	☐	☐	☐	☐
Dysgeusia (altered taste)	☐	☐	☐	☐
Sore throat	☐	☐	☐	☐
Throat sensitivity	☐	☐	☐	☐
Cough	☐	☐	☐	☐
Sputum production	☐	☐	☐	☐
Hoarse voice/voice change	☐	☐	☐	☐

	No alert	Alert if symptom is mild	Alert if moderate	Alert if severe
Hemoptysis	☐	☐	☐	☐
Avoid certain food and/or drink	☐	☐	☐	☐
Difficulty swallowing/choking	☐	☐	☐	☐
Aphonia	☐	☐	☐	☐
Dry mouth	☐	☐	☐	☐
Ear pain	☐	☐	☐	☐
New hearing loss	☐	☐	☐	☐
Tinnitus	☐	☐	☐	☐
Joint pain	☐	☐	☐	☐
Joint swelling	☐	☐	☐	☐
Joint stiffness	☐	☐	☐	☐
Muscle weakness	☐	☐	☐	☐
Muscle pain (myalgia)	☐	☐	☐	☐
Abdominal pain	☐	☐	☐	☐
Nausea	☐	☐	☐	☐
Vomiting	☐	☐	☐	☐
Diarrhoea	☐	☐	☐	☐
Gastritis (indigestion)	☐	☐	☐	☐
Loss of appetite	☐	☐	☐	☐
Weight loss/anorexia	☐	☐	☐	☐
Constipation	☐	☐	☐	☐
Abdominal bloating	☐	☐	☐	☐
Skipping meals	☐	☐	☐	☐
Bowel incontinence	☐	☐	☐	☐
Ulcer	☐	☐	☐	☐
Depression	☐	☐	☐	☐
Anxiety	☐	☐	☐	☐
Mood change	☐	☐	☐	☐

	No alert	Alert if symptom is mild	Alert if moderate	Alert if severe
Self-harm/Suicidal ideation*	☐	☐	☐	☐
Mood swings	☐	☐	☐	☐
Stress	☐	☐	☐	☐
Loneliness	☐	☐	☐	☐
Not same person	☐	☐	☐	☐
Memory loss (remembering things)	☐	☐	☐	☐
Difficulty thinking	☐	☐	☐	☐
Difficulty planning	☐	☐	☐	☐
Brain fog	☐	☐	☐	☐
Inability to concentrate	☐	☐	☐	☐
Difficulty focussing on a single task	☐	☐	☐	☐
Disorientation	☐	☐	☐	☐
Sleep disorder (eg insomnia)	☐	☐	☐	☐
neuraglia/ neuropathy	☐	☐	☐	☐
needle pains arms & legs (parathesia)	☐	☐	☐	☐
Tremors	☐	☐	☐	☐
Seizures	☐	☐	☐	☐
Balance problems	☐	☐	☐	☐
Cannot fully control movement	☐	☐	☐	☐
Difficulty with communcation	☐	☐	☐	☐
Wordfinding difficulties	☐	☐	☐	☐
Hallucinations	☐	☐	☐	☐
Physical slowing down	☐	☐	☐	☐
Fatigue	☐	☐	☐	☐
Hair loss	☐	☐	☐	☐
Itchy skin/pruritus	☐	☐	☐	☐
Skin rash	☐	☐	☐	☐
Spots on feet	☐	☐	☐	☐

	No alert	Alert if symptom is mild	Alert if moderate	Alert if severe
Hives	☐	☐	☐	☐
Random bruising	☐	☐	☐	☐
Dry skin	☐	☐	☐	☐
Fever	☐	☐	☐	☐
Chills	☐	☐	☐	☐
Sweats	☐	☐	☐	☐
Hot flushes	☐	☐	☐	☐
Headache	☐	☐	☐	☐
Dizzines/Vertigo	☐	☐	☐	☐
Unspecified pain/body ache	☐	☐	☐	☐
Bladder incontinence	☐	☐	☐	☐
Red eyes	☐	☐	☐	☐
Dry eyes	☐	☐	☐	☐
Itchy eyes	☐	☐	☐	☐
Vision disturbances	☐	☐	☐	☐
Floaters*	☐	☐	☐	☐
Flashing lights	☐	☐	☐	☐
Blurry vision*	☐	☐	☐	☐
Pressure behind the eyes	☐	☐	☐	☐
Sensitivity to light	☐	☐	☐	☐

Please provide any further information to help us develop the alerting thresholds for the SBQ™-LC-LC.

16. Symptom Clusters

15. Are there specific clusters of symptoms that would warrant concern (e.g. for a stroke)? Please list symptoms and indicate why this cluster would be cause for concern. *

Review copies can only be used for the limited purpose of examining the suitability of the questionnaire for subsequent research and/or clinical use and cannot be used in research or in clinical practice or distributed to others. You are not authorized to modify, retype, translate, copy or otherwise duplicate the questionnaire except with the further and prior written permission of the developers / copyright holders / distributors. Some content has been deleted in this review version.

BREATHING

These questions are about your BREATHING symptoms. For each question, please choose the response that best describes your experience over the last 7 days.

In the last 7 days, how severe was your **shortness of breath (difficulty breathing) when sitting** at its worst?

- ☐ 0 - None
- ☐ 1 - Mild
- ☐ 2 - Moderate
- ☐ 3 - Severe

In the last 7 days, how severe was your **shortness of breath (difficulty breathing) when climbing a flight of stairs** at its worst?

****Response scale removed****

In the last 7 days, how severe was your **shortness of breath (difficulty breathing) when lying flat** at its worst?

- ☐ 0 - None
- ☐ 1 - Mild
- ☐ 2 - Moderate
- ☐ 3 - Severe

Review Copy Only - Not for Use

Please go to the next page

In the last 7 days, did you **wake up at night short of breath**?

- ☐ 0 - No
- ☐ 1 - Yes

In the last 7 days, was your **breathing faster than usual**?

- ☐ 0 - No
- ☐ 1 - Yes

In the last 7 days, how severe was the **tightness of your chest** at its worst?

****Response scale removed****

In the last 7 days, how severe was your **wheezing (noisy breathing)** at its worst?

- ☐ 0 - None
- ☐ 1 - Mild
- ☐ 2 - Moderate
- ☐ 3 - Severe

**Breathing Scale Raw Score:**	

Review Copy Only - Not for Use

Category Probability Curves

For each graph, the y-axis represents the expected probability of endorsement of any given category when a person responds to the item. The x-axis represents the person ability relative to the item difficulty, with origins set to 0. The scale is measured in logits. Locations to the right of ‘0’ represent greater and greater levels of symptom burden. Moving to the left of ‘0’ locations represent lower levels of symptom burden. Categories should advance in ascending order from left to right along the x-axis and each have a distinct peak (indicating that the category is the most probable (modal) category at that point on the latent variable). The crossover points between two curves are the equal probability points of thresholds. Each category curved is colour coded: category 0 = red, category 1 = blue; category 2 = pink; category 3-4 = dark green.

Category Probability Curves – Breathing

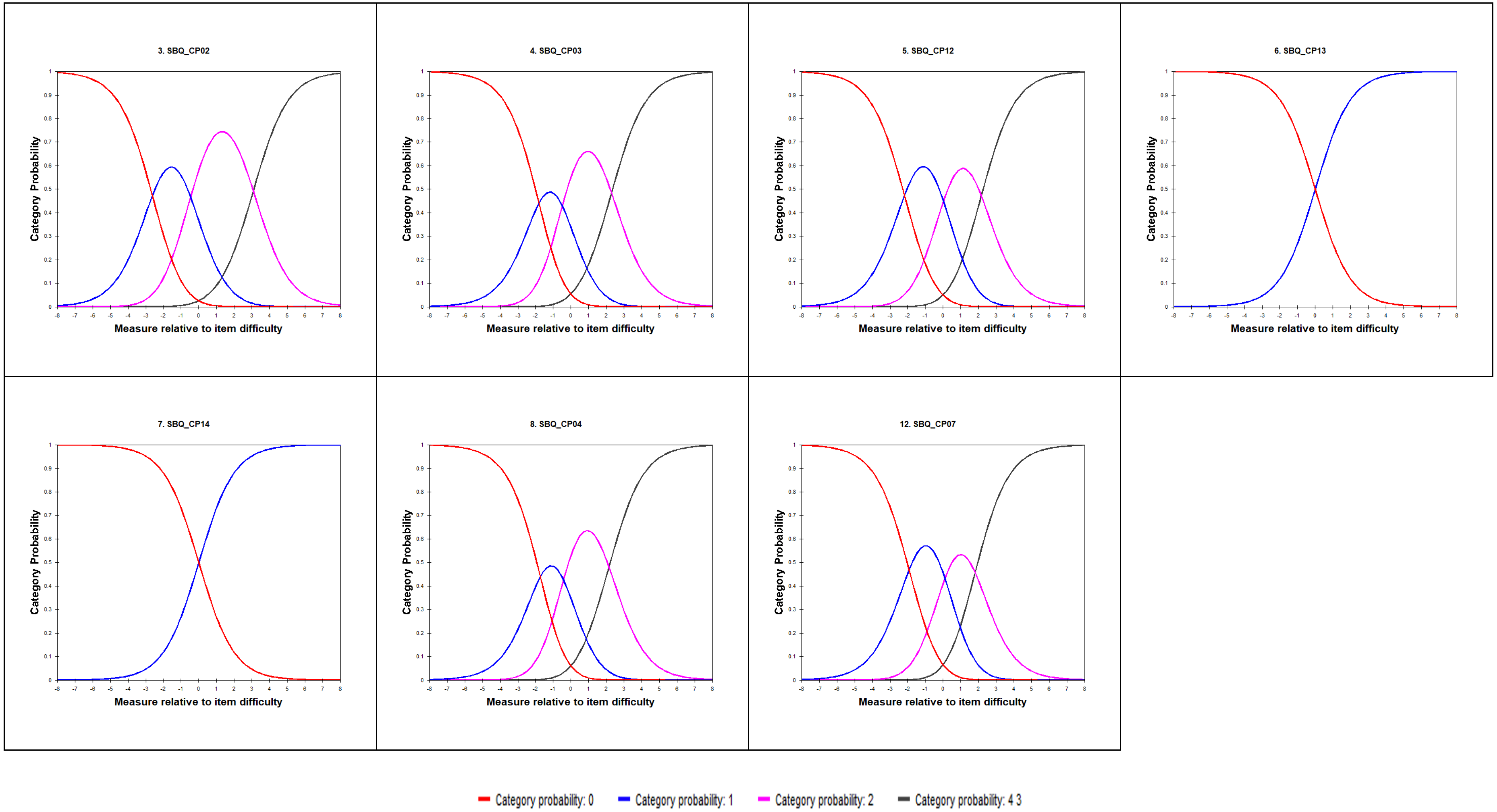

Category Probability Curves – Pain

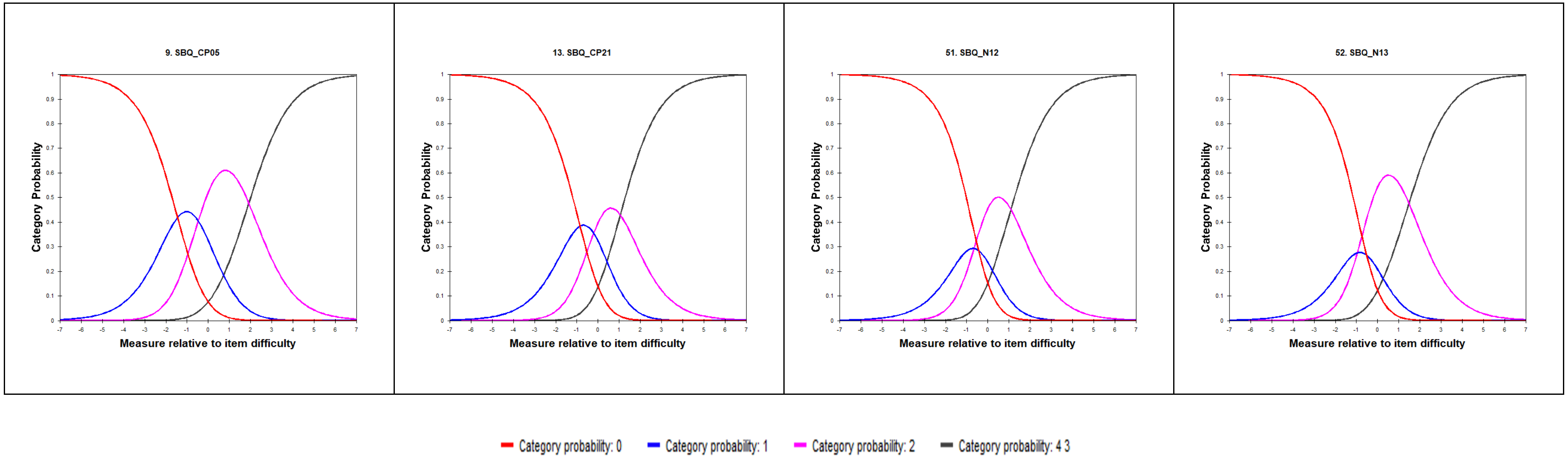

Category Probability Curves – Circulation

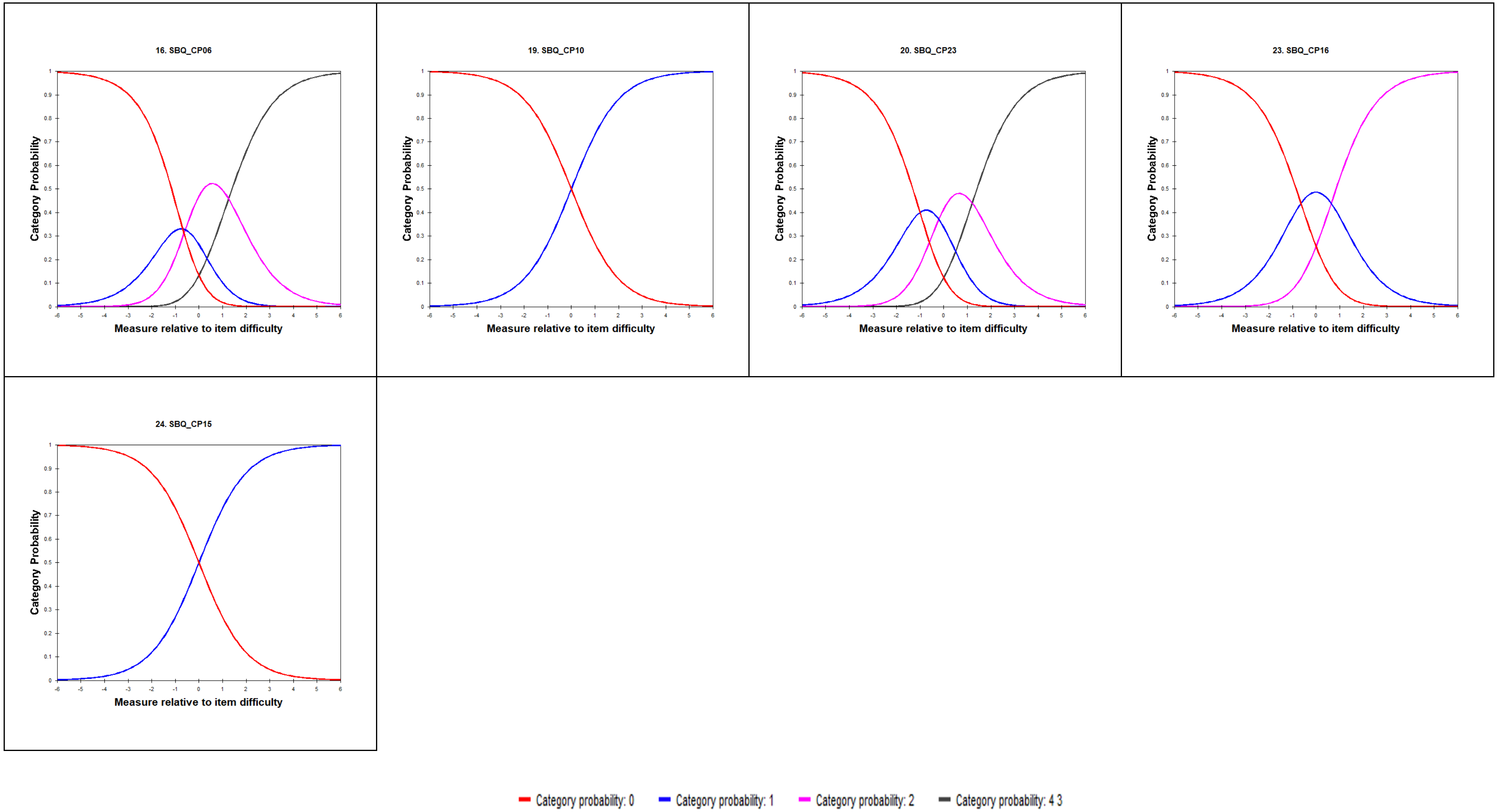

Category Probability Curves – Fatigue

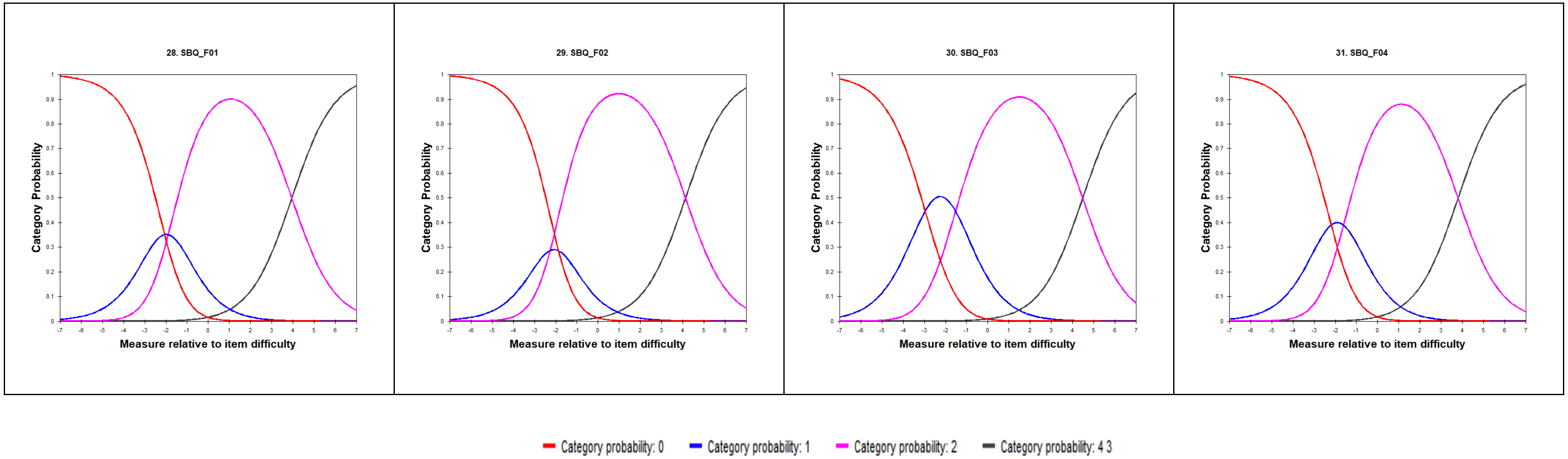

Category Probability Curves – Memory, Thinking and Communication

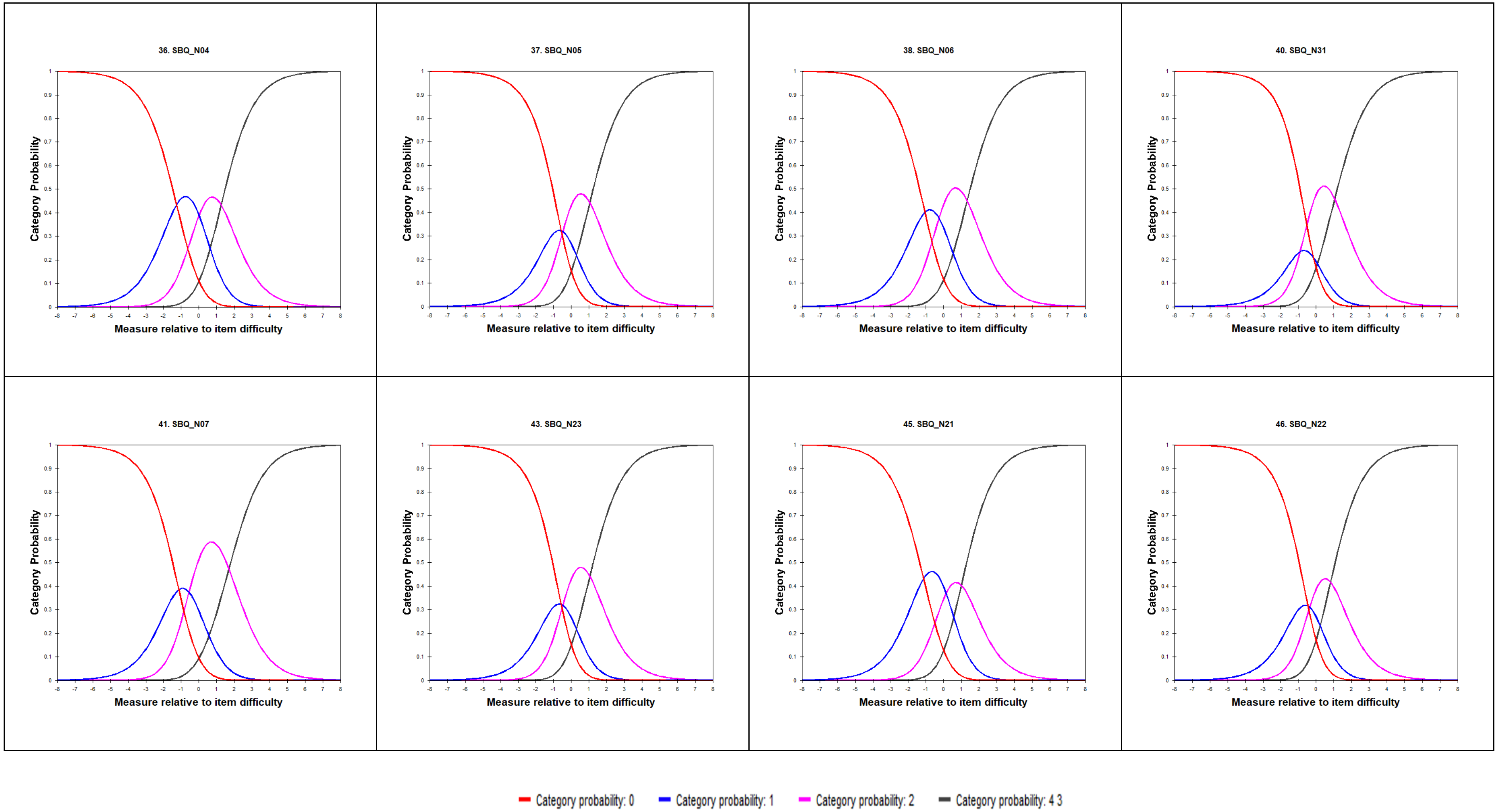

Category Probability Curves – Memory, Thinking and Communication continued...

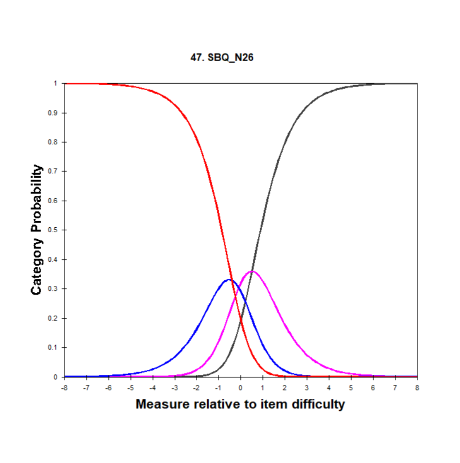

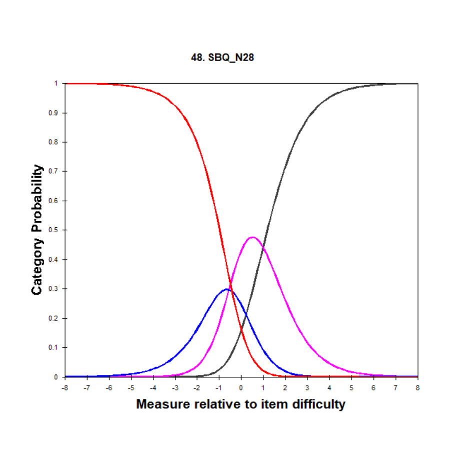

Category probability: 0   Category probability: 1   Category probability: 2   Category probability: 4 3

Category Probability Curves – Movement

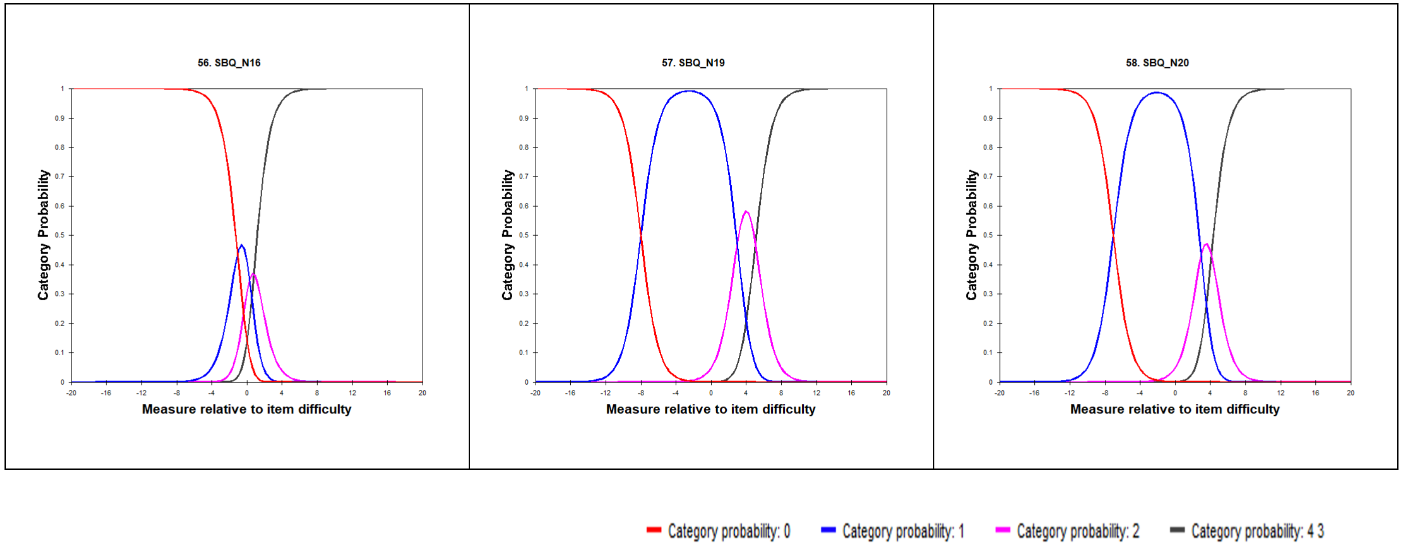

Category Probability Curves – Sleep

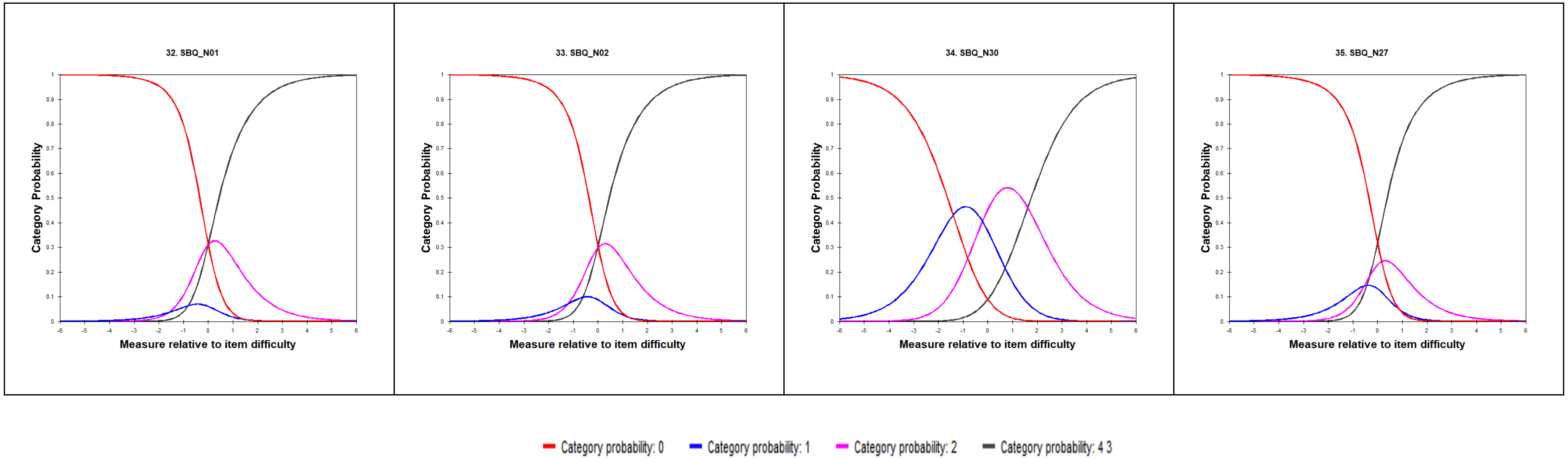

Category Probability Curves – Ear, Nose and Throat

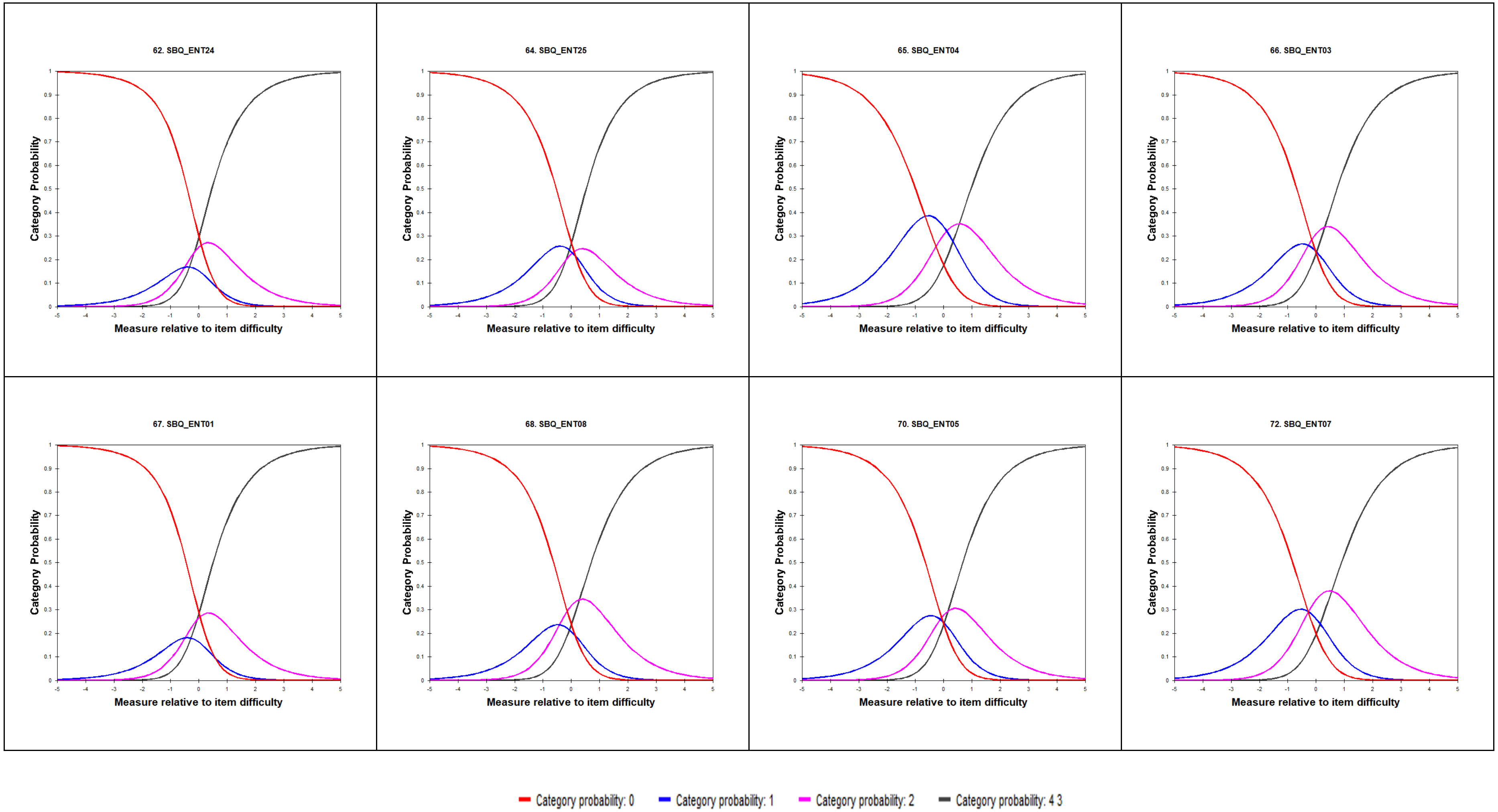

Category Probability Curves: Ear, Nose and Throat continued....

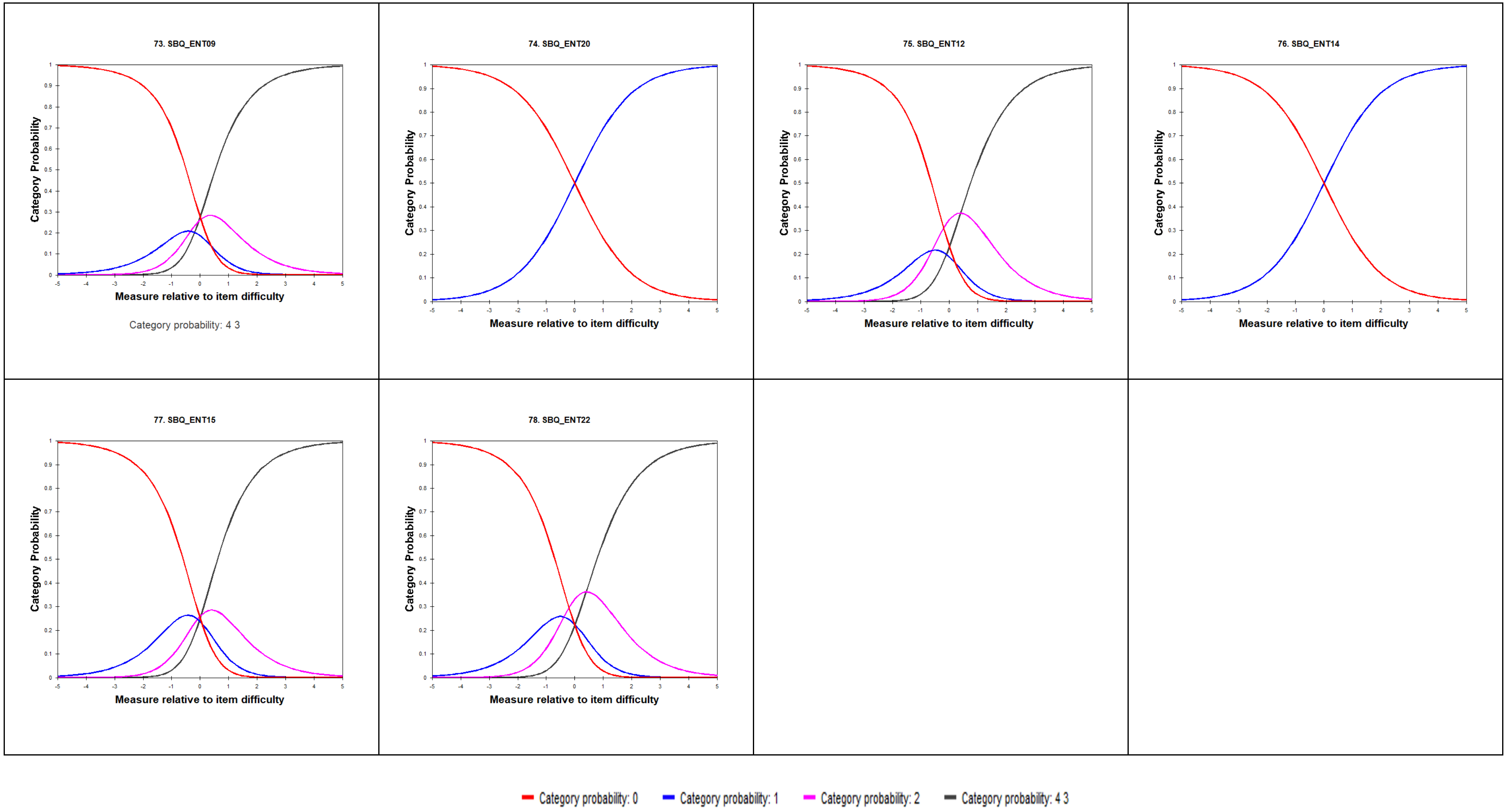

Category Probability Curves – Stomach and Digestion

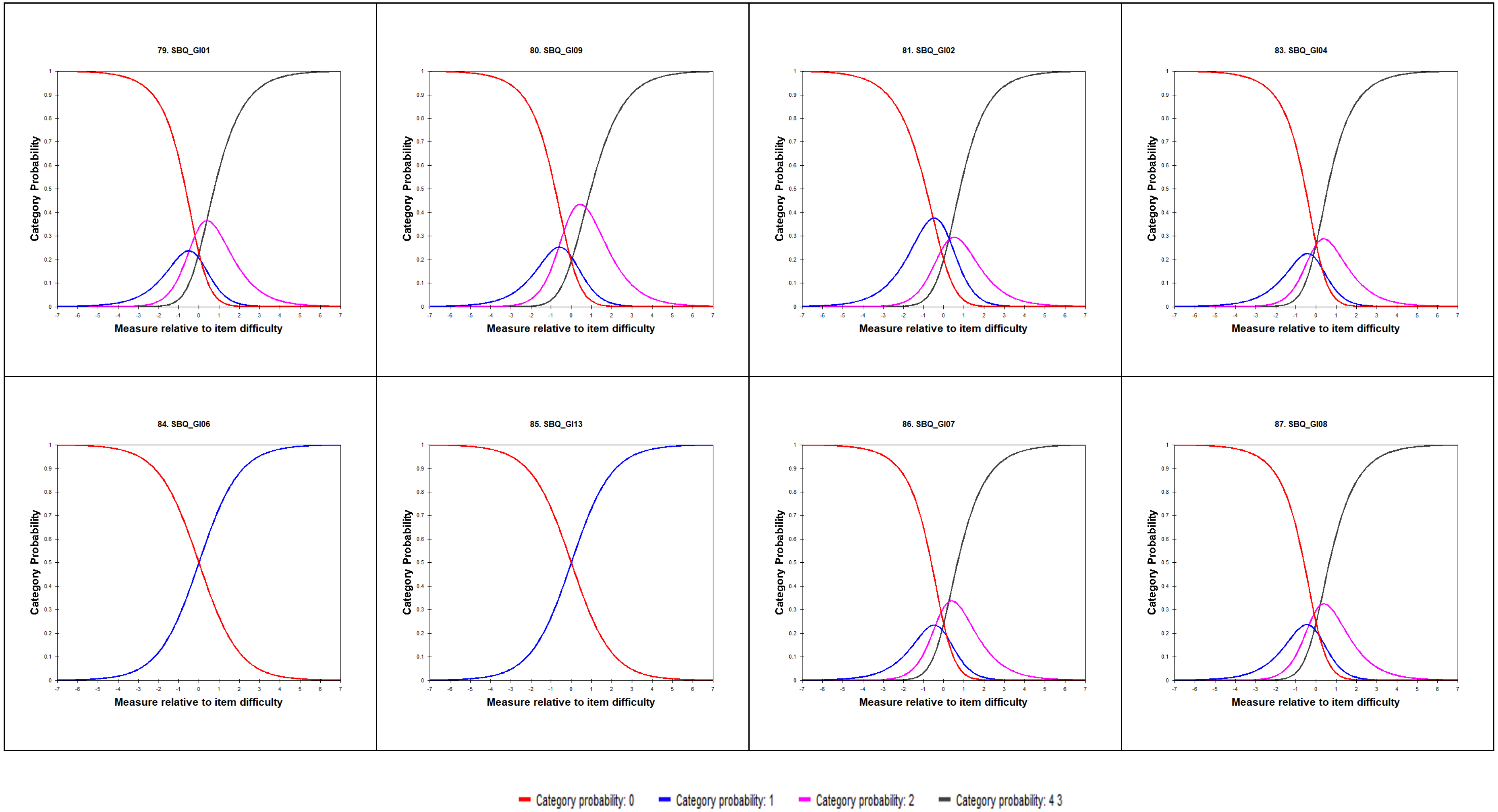

Category Probability Curves – Muscles and Joints

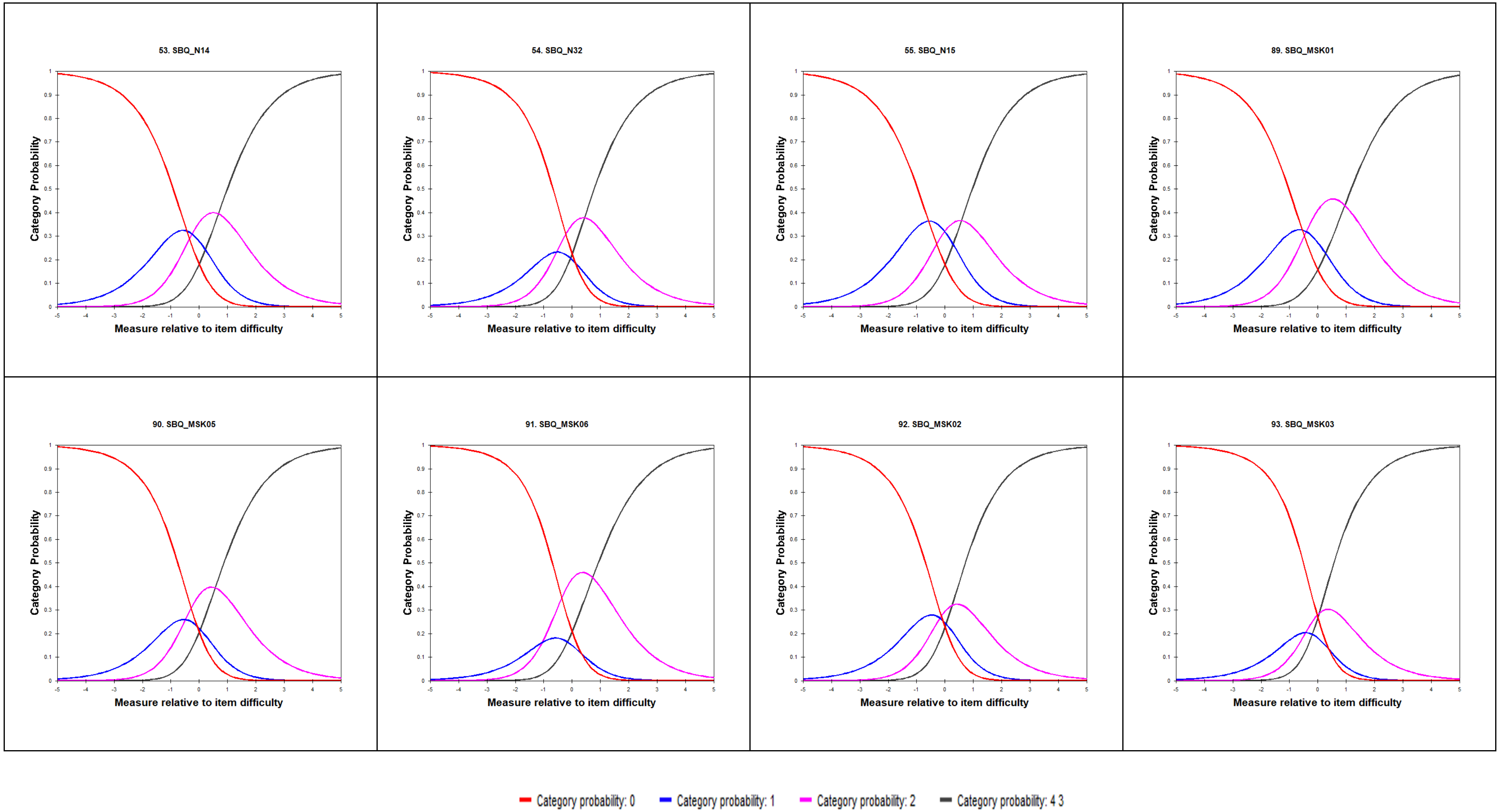

Category Probability Curves – Muscles and Joints continued...

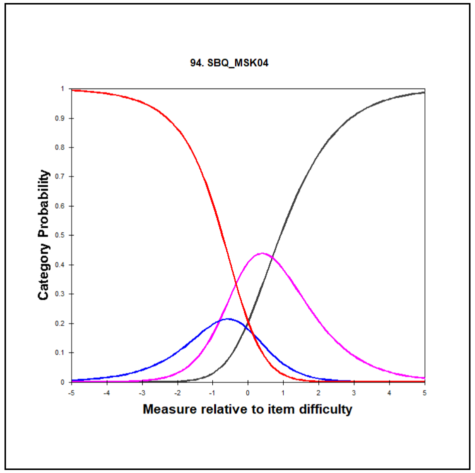

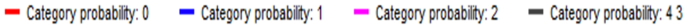

Category Probability Curves – Mental Health and Wellbeing

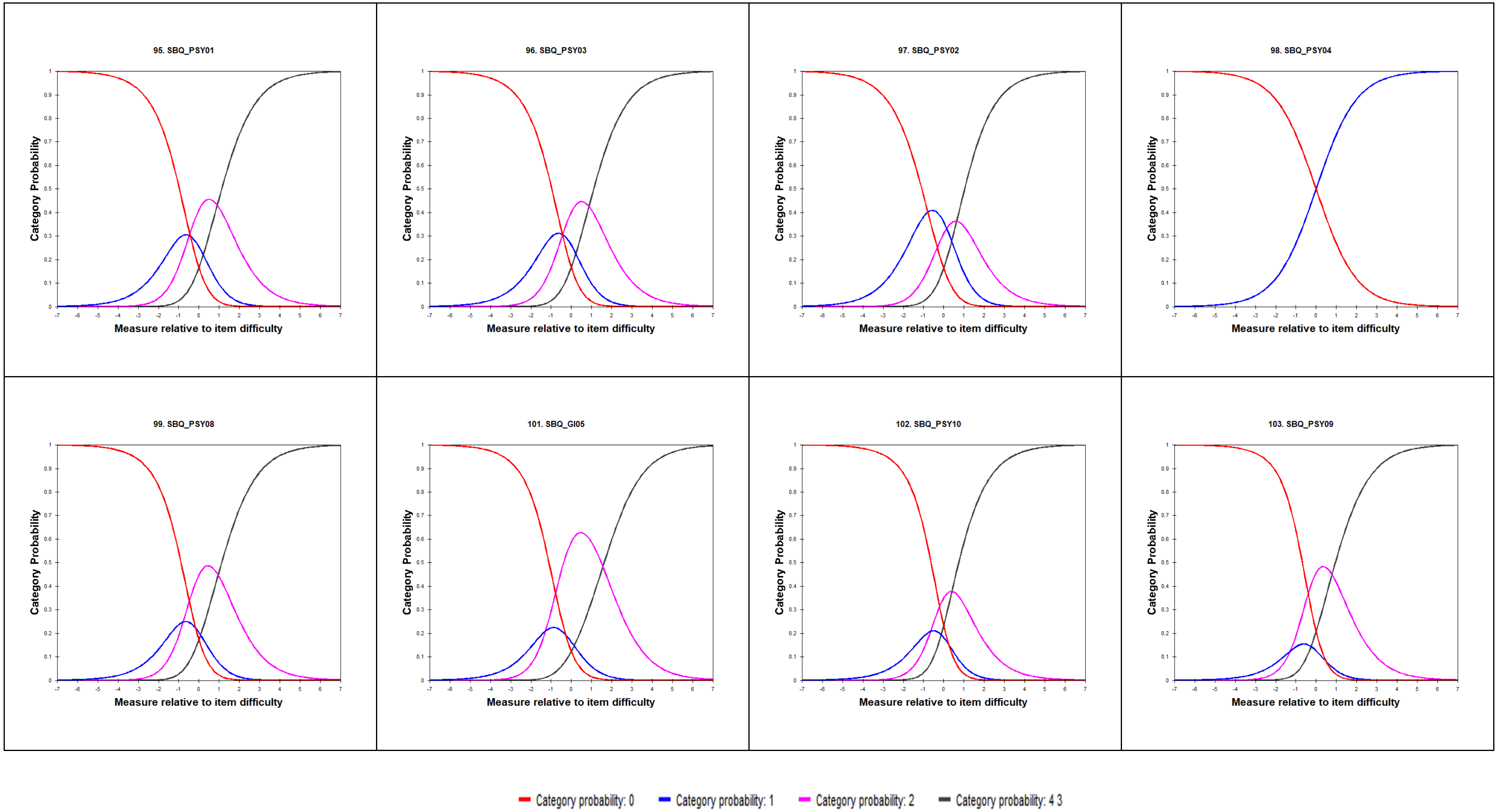

Scale: Mental Health and Wellbeing continued...

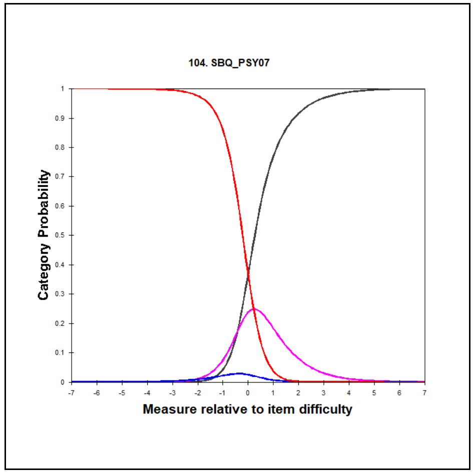

Category probability: 0 Category probability: 1 Category probability: 2 Category probability: 4 3

Category Probability Curves – Skin and Hair

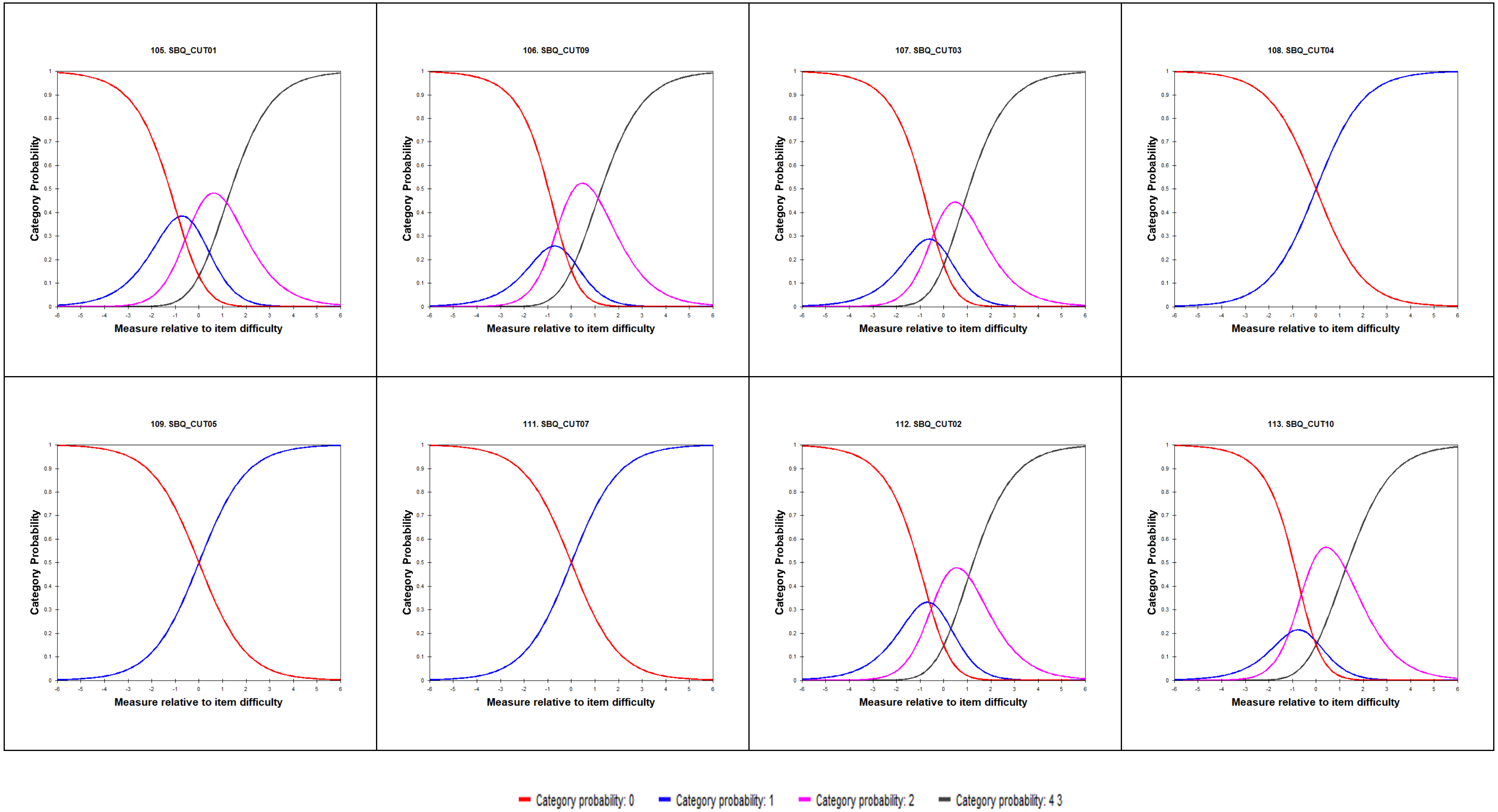

Category Probability Curves – Eyes

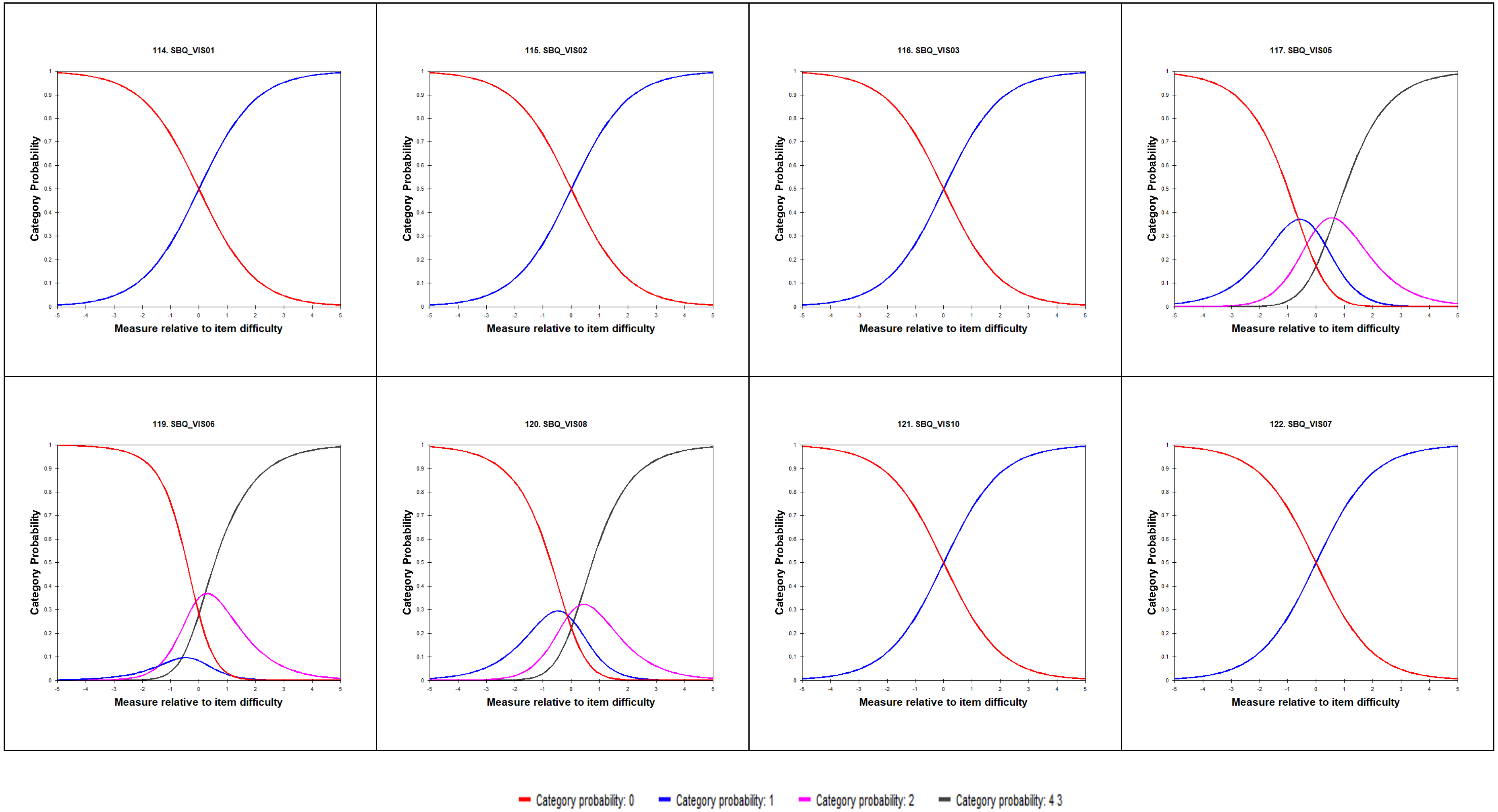

Category Probability Curves – Eyes

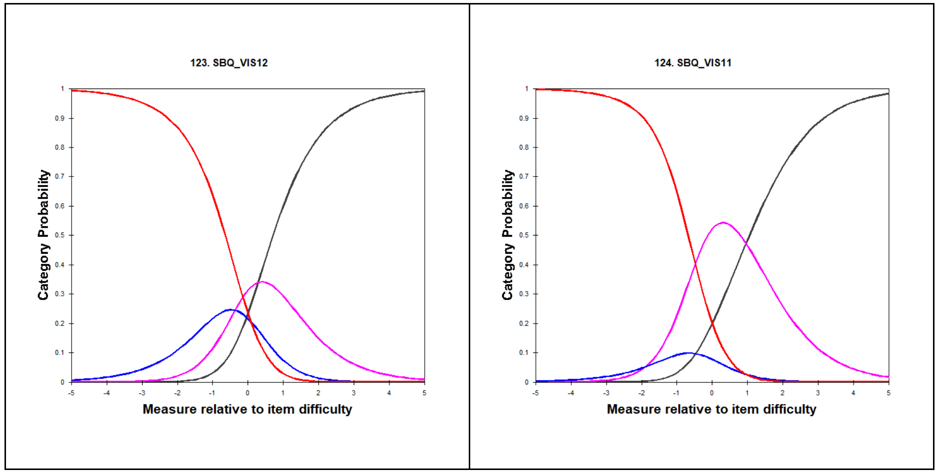

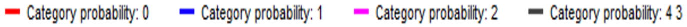

Category Probability Curves – Female Reproductive and Sexual Health

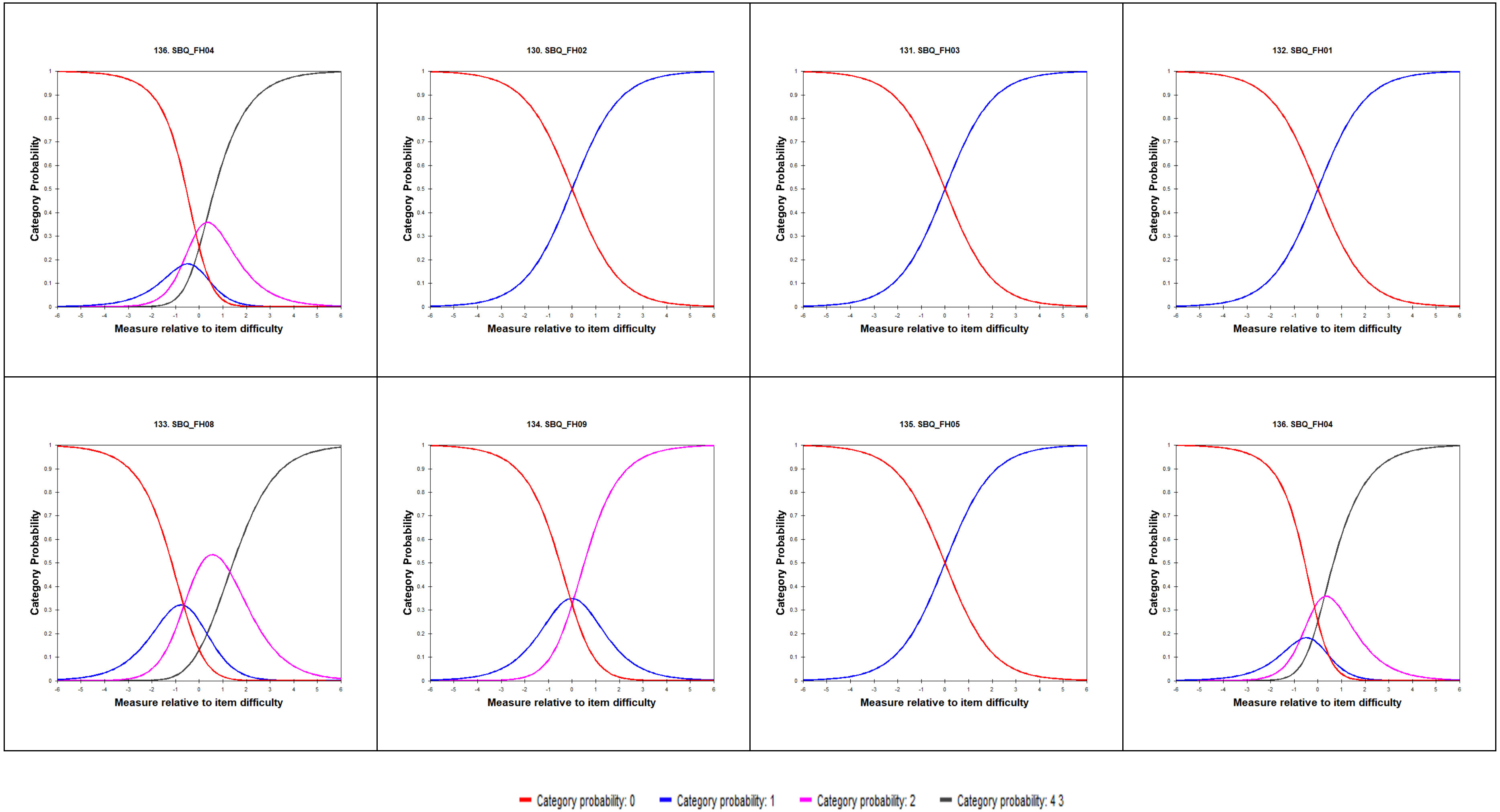

Category Probability Curves – Male Reproductive and Sexual Health

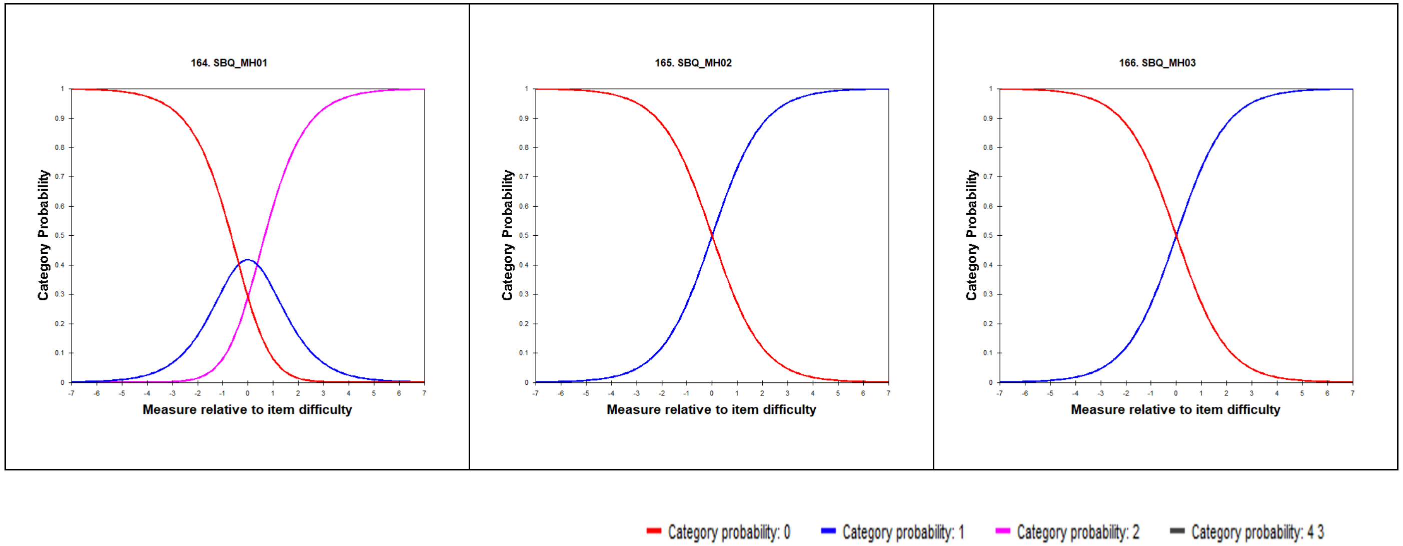

Category Probability Curves – Other Symptoms

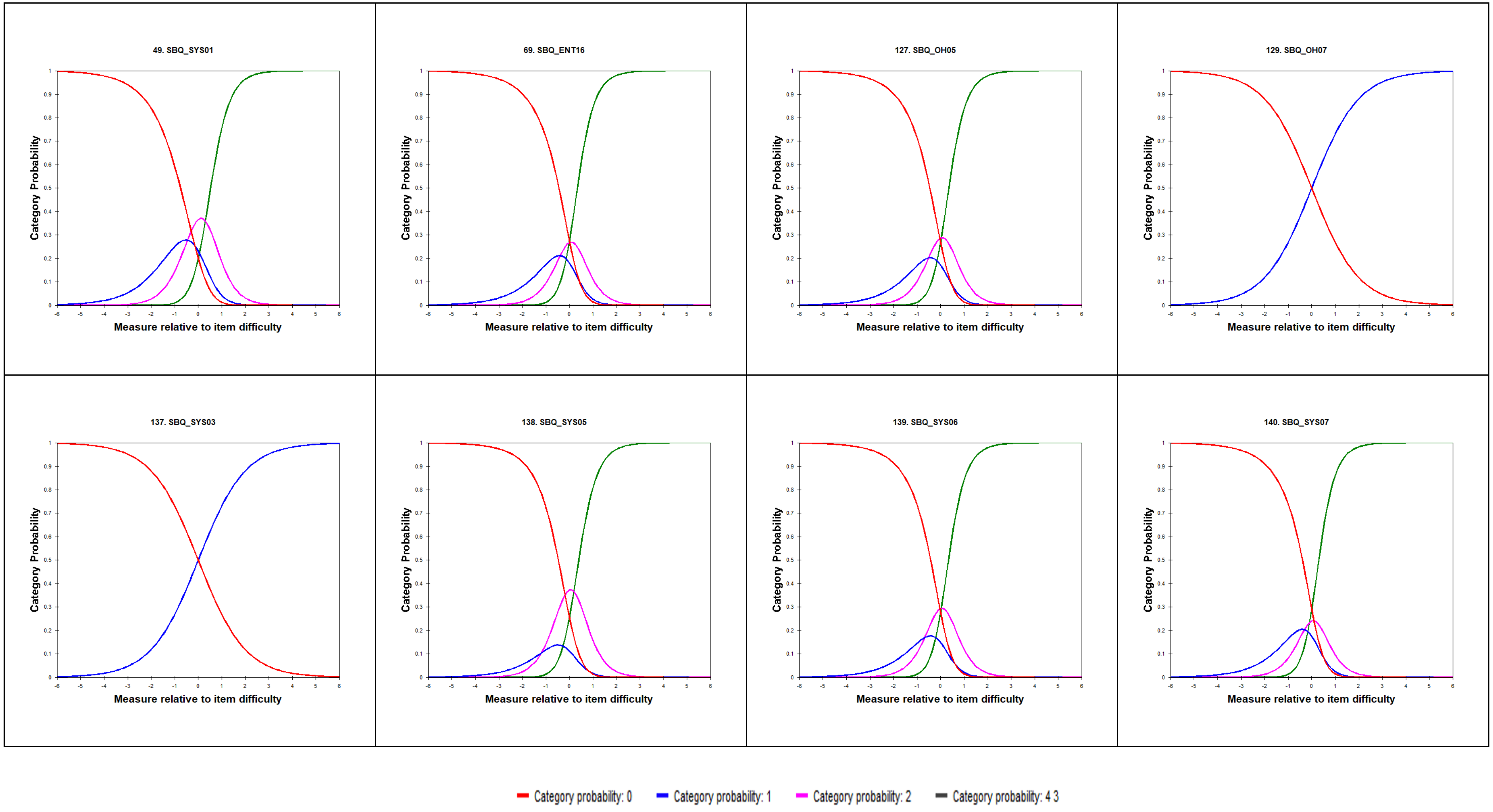

Category Probability Curves – Other symptoms continued...

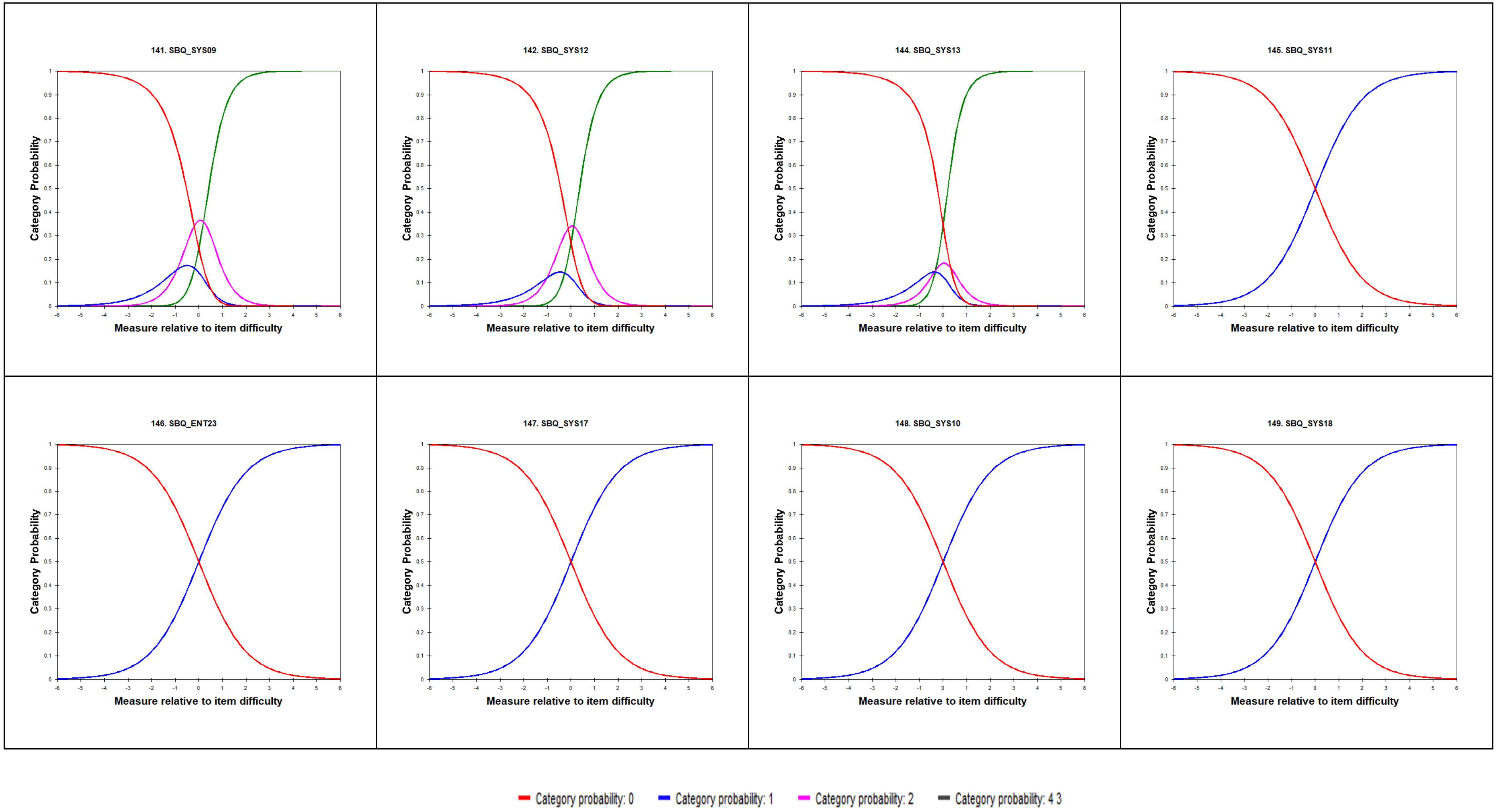

Category Probability Curves – Other symptoms continued...

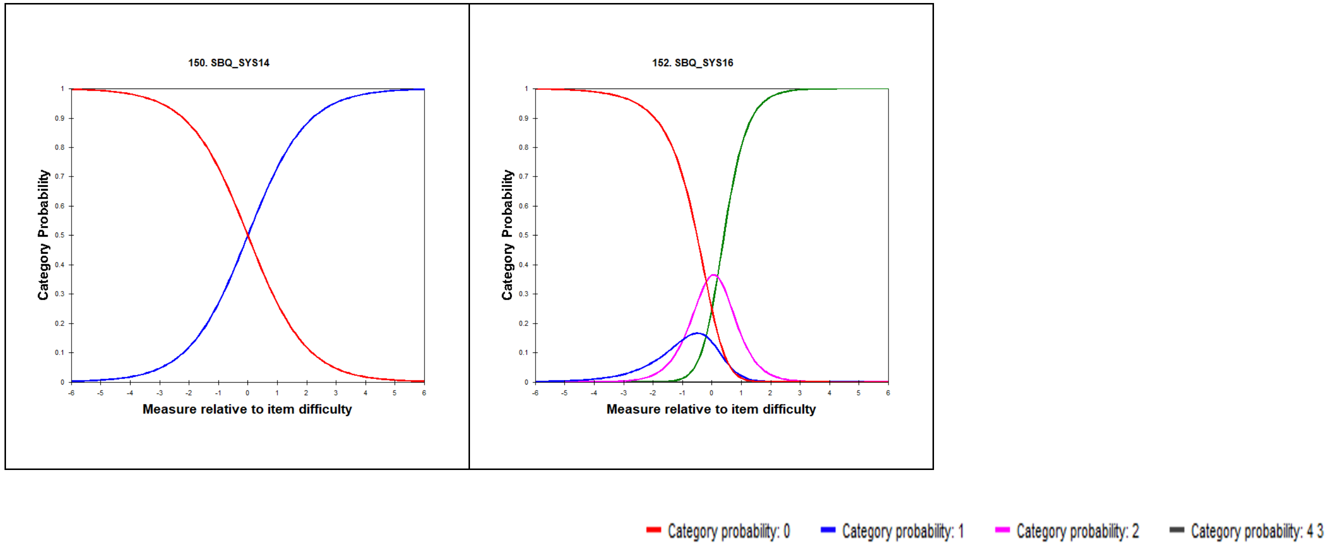

Category Probability Curves – Interference

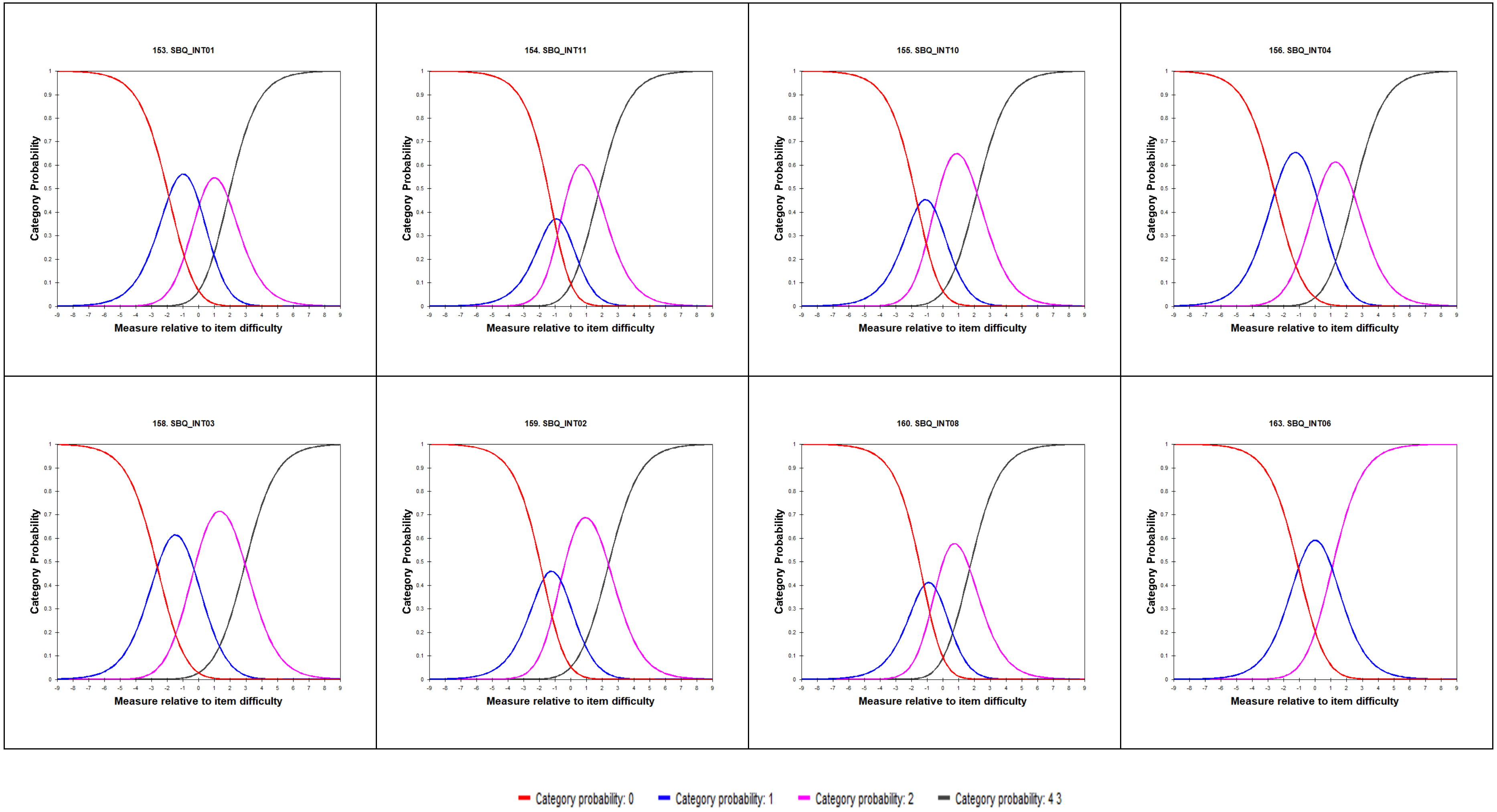

S2 Figure: Item-Person Maps for the SBQ™-LC (Version 1.0)

For each scale, the person-item map displays the location of person abilities and item difficulties respectively along the same latent dimension (y-axis).

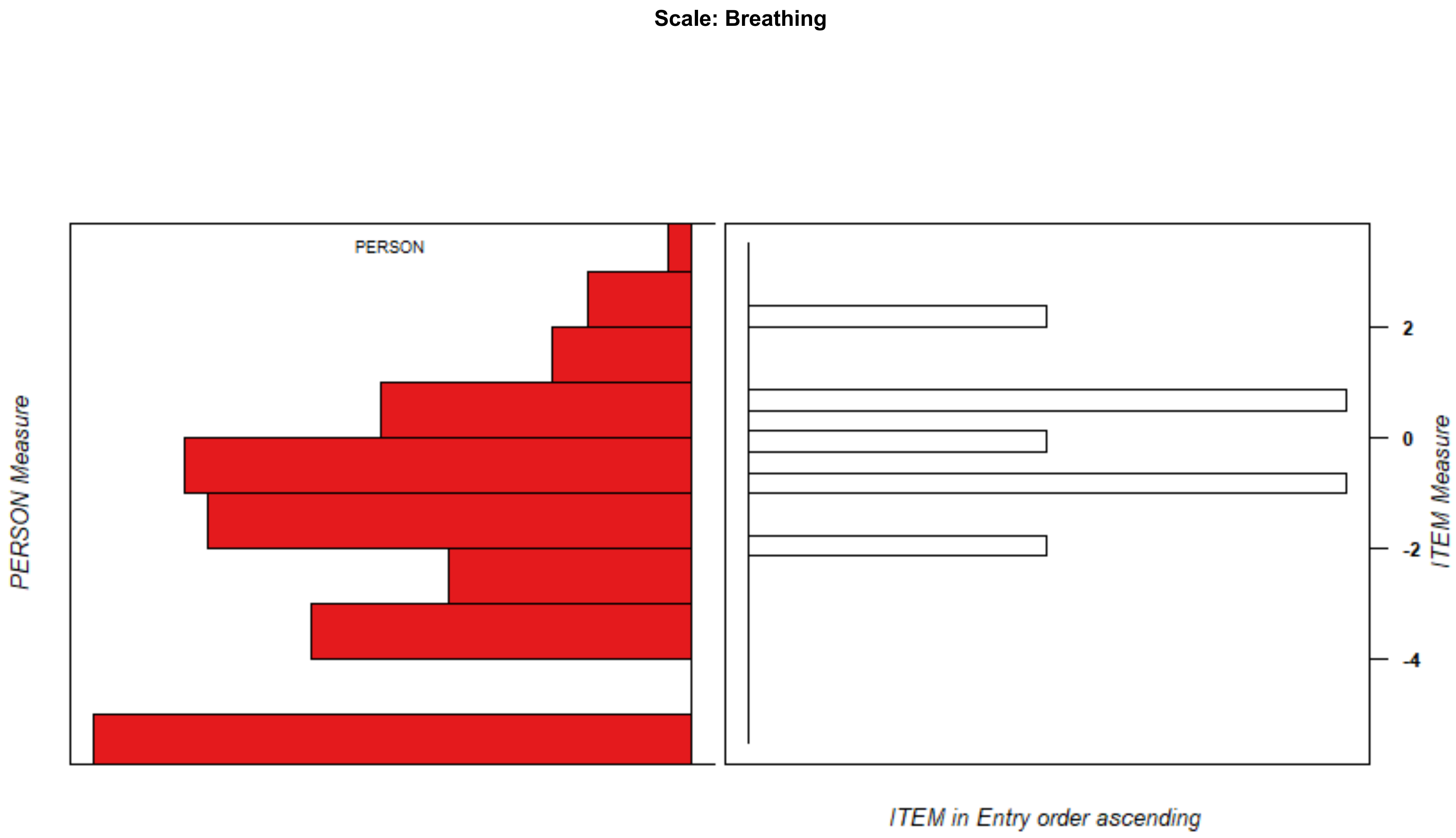

Scale: Breathing
PERSON
PERSON Measure
2
0
-2
-4
ITEM Measure
ITEM in Entry order ascending

Scale: Pain

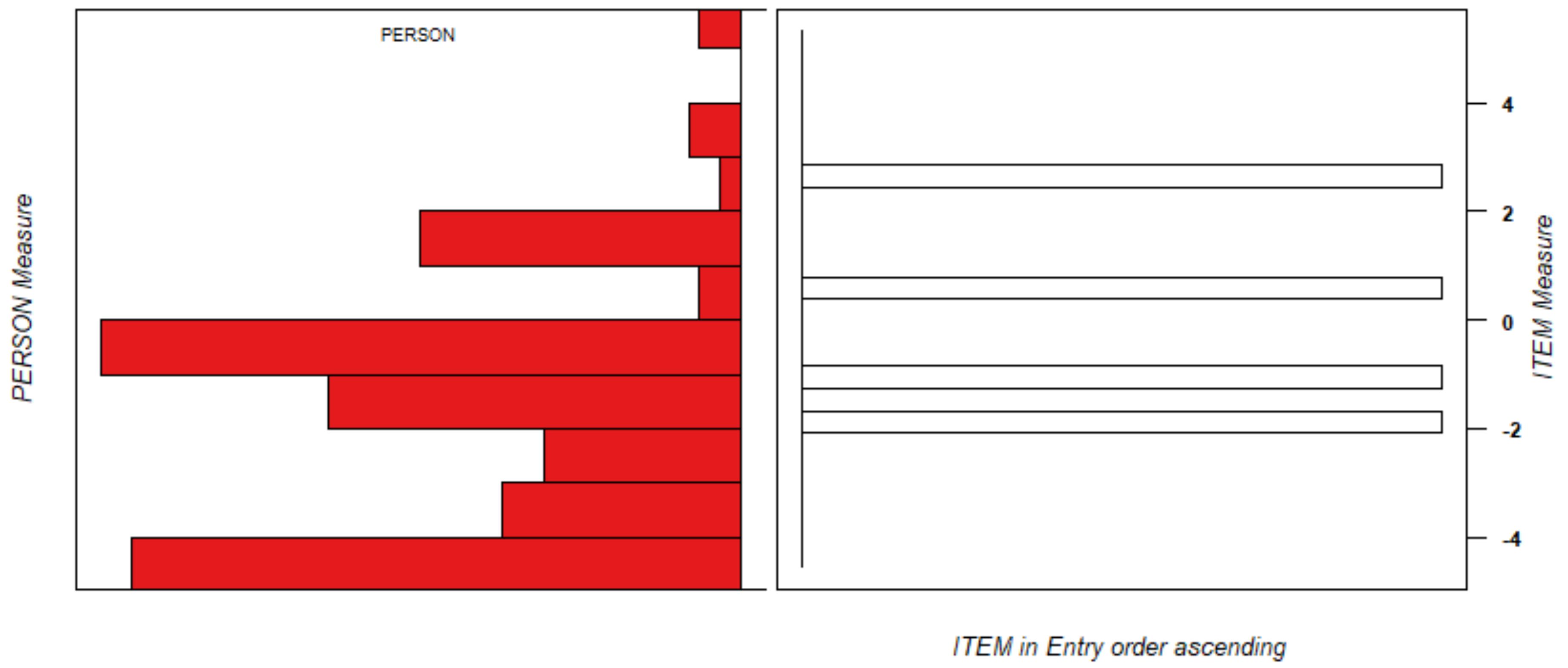
PERSON
PERSON Measure
4
2
0
-2
-4
ITEM Measure
ITEM in Entry order ascending

Scale: Circulation

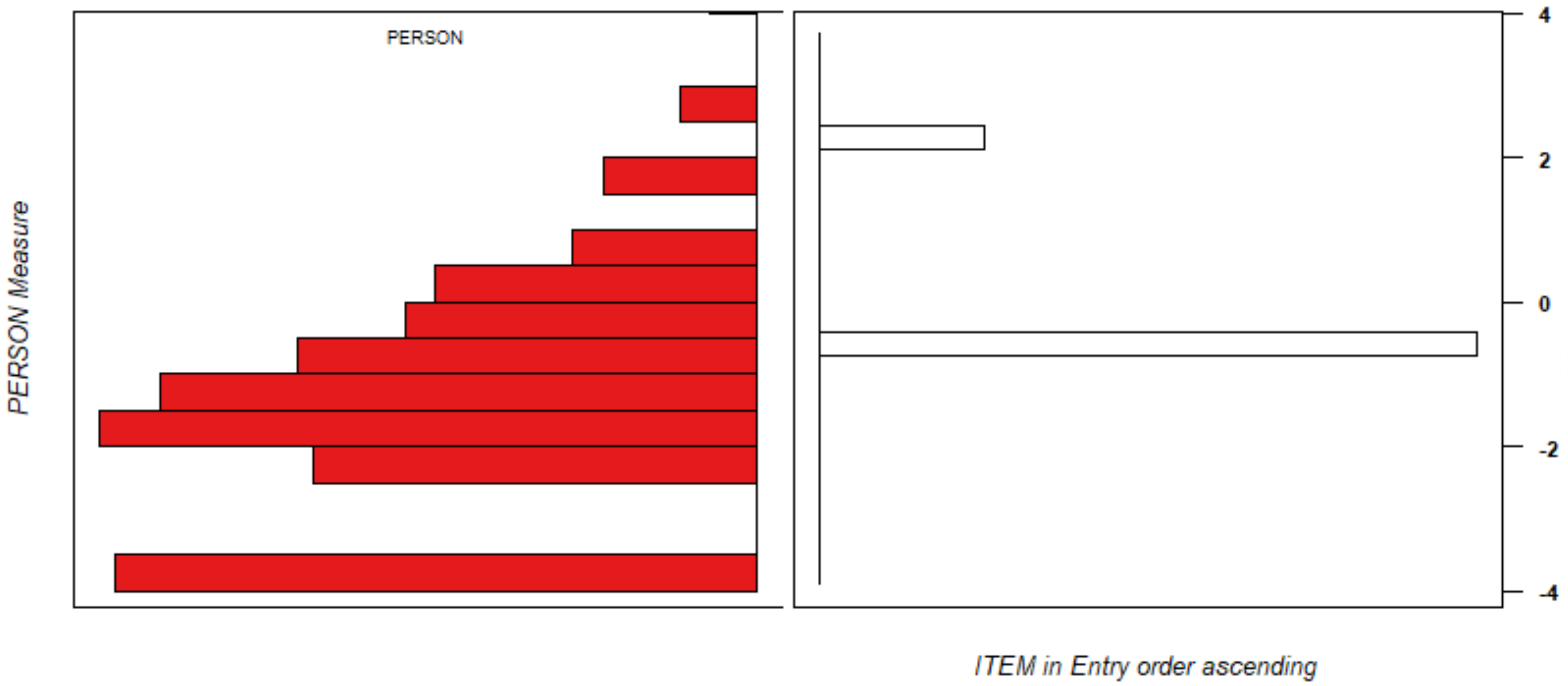

Scale: Fatigue

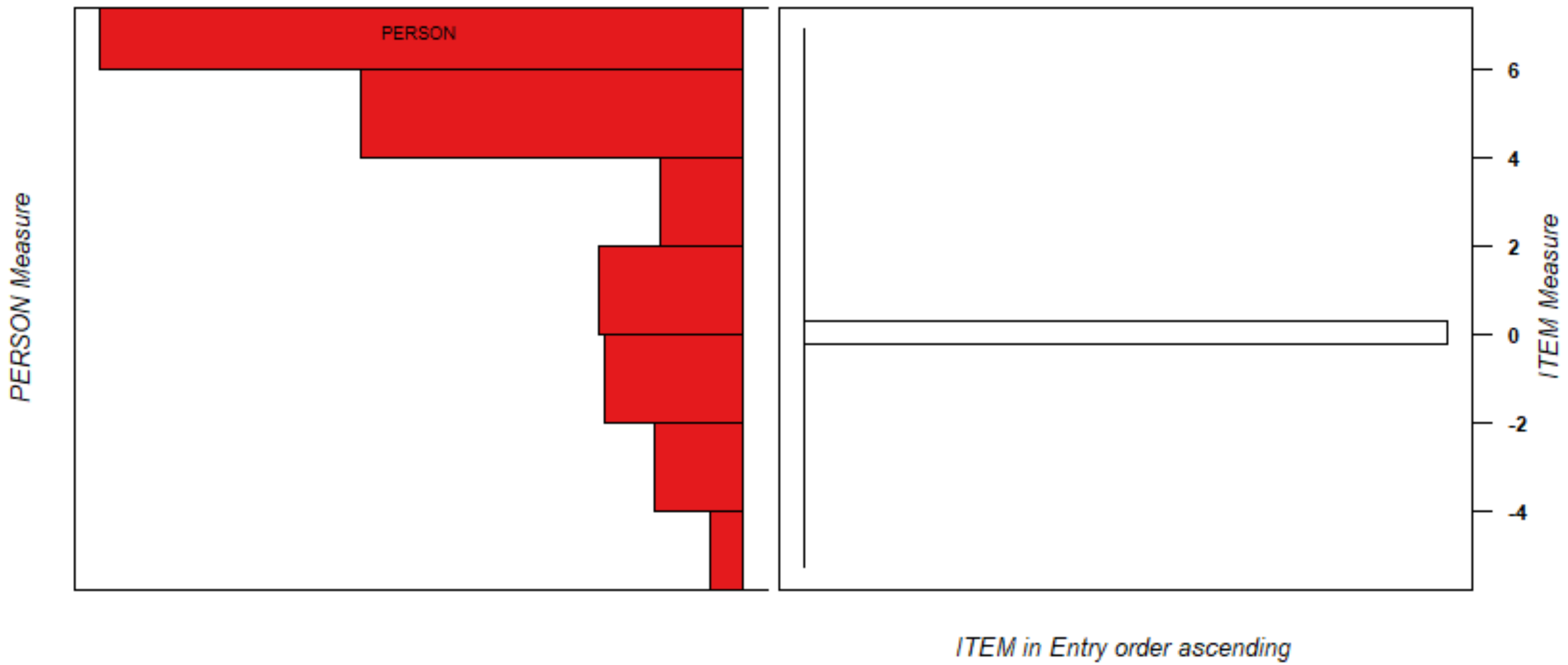

Scale: Memory, Thinking & Communication

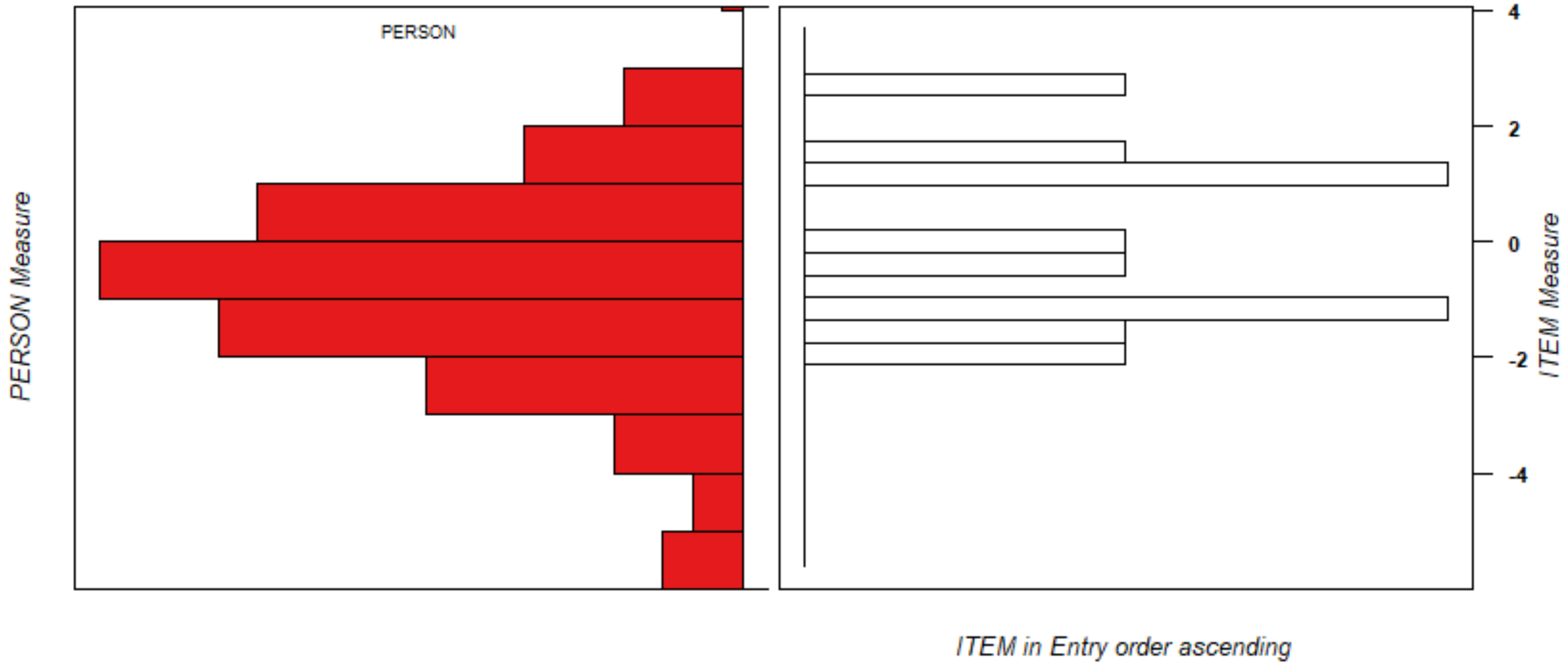

Scale: Movement

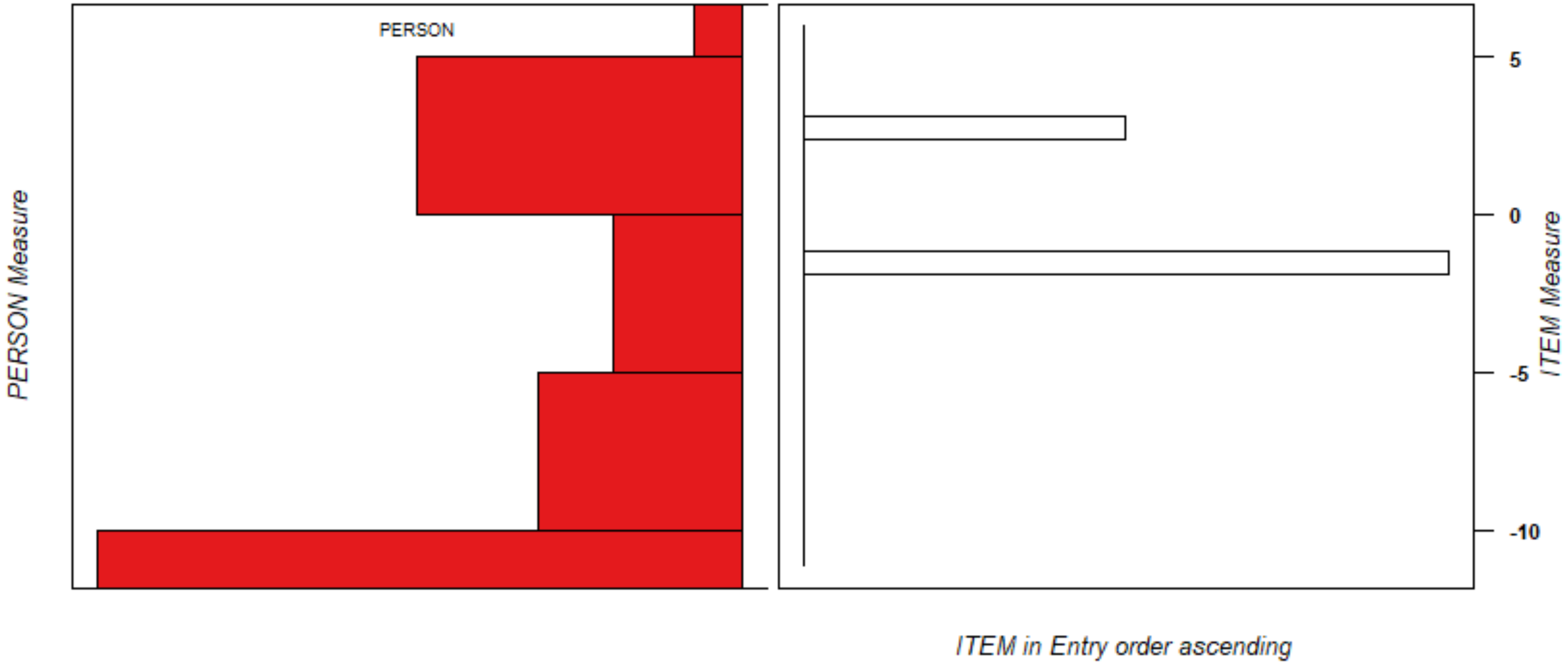

Scale: Sleep

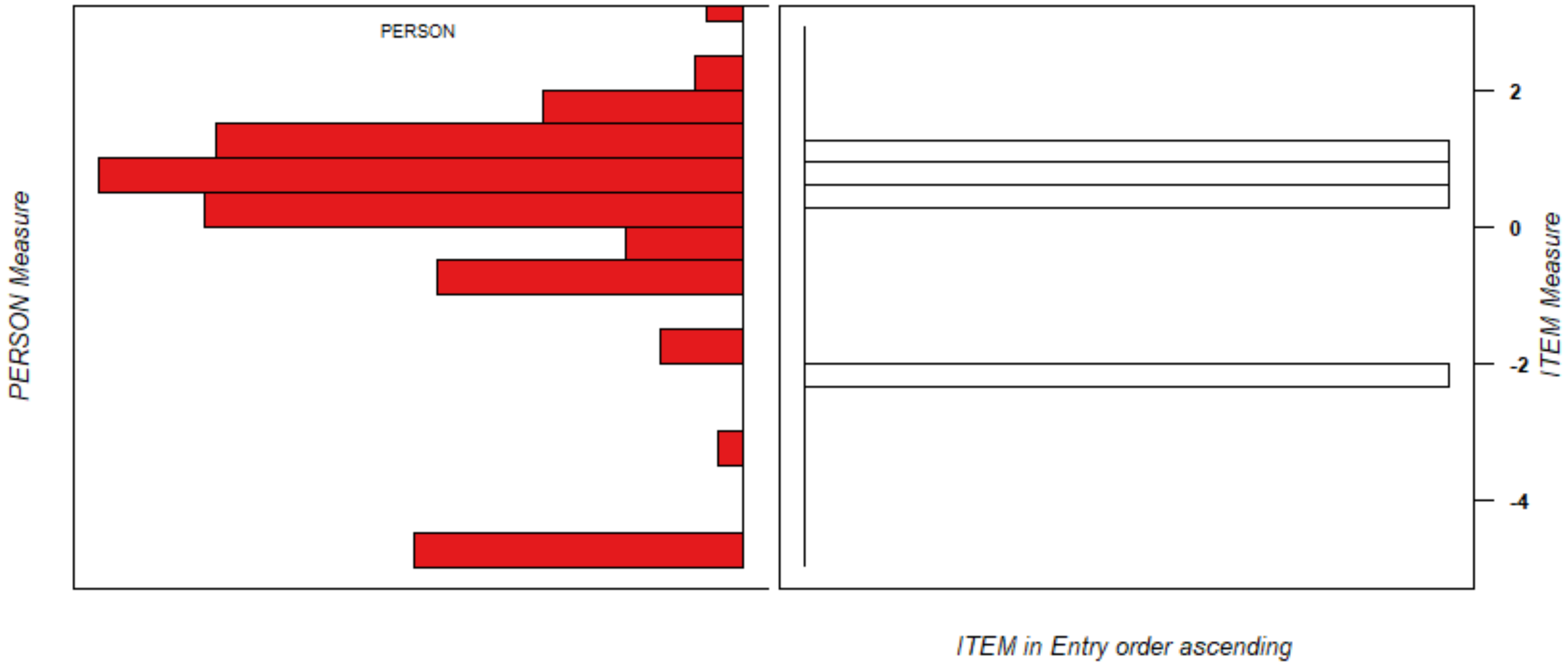
PERSON
PERSON Measure
2
0
-2
-4
ITEM Measure
ITEM in Entry order ascending

Scale: Ear, Nose & Throat

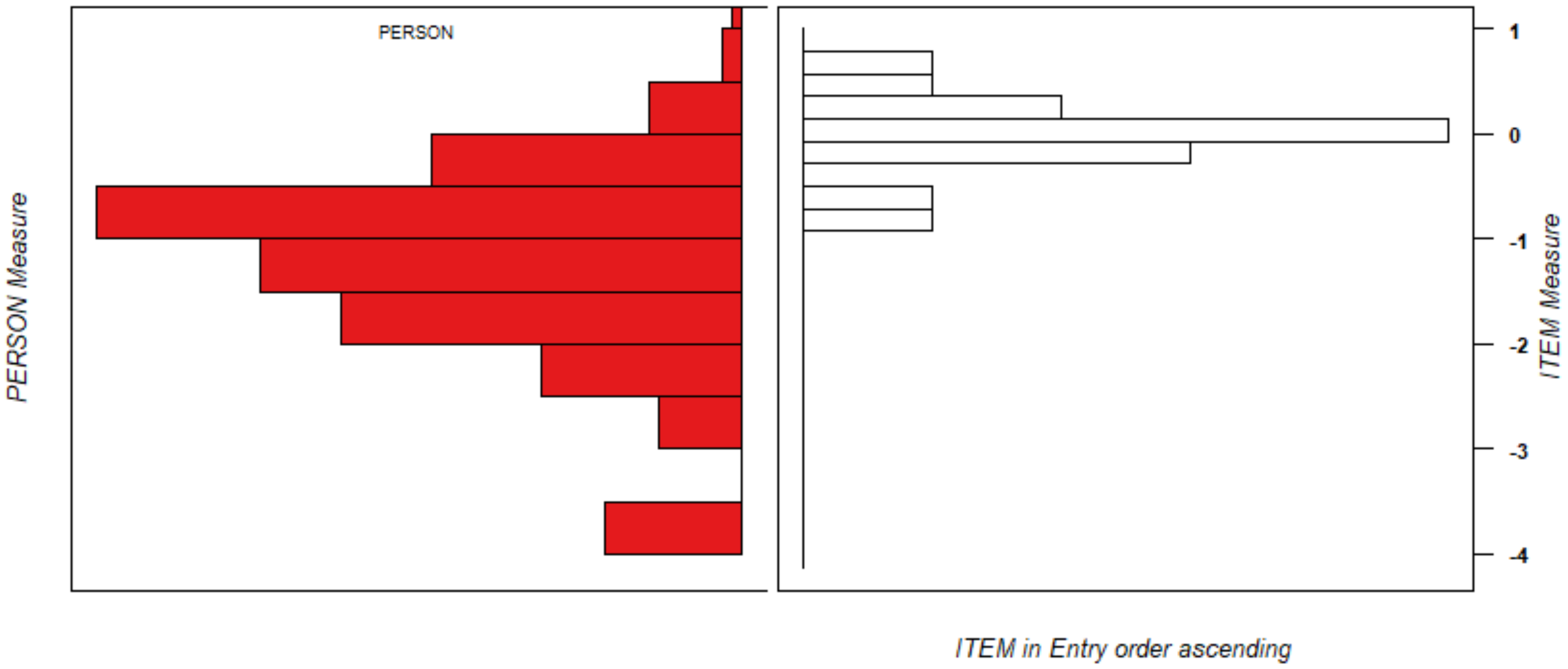

Scale: Stomach and Digestion

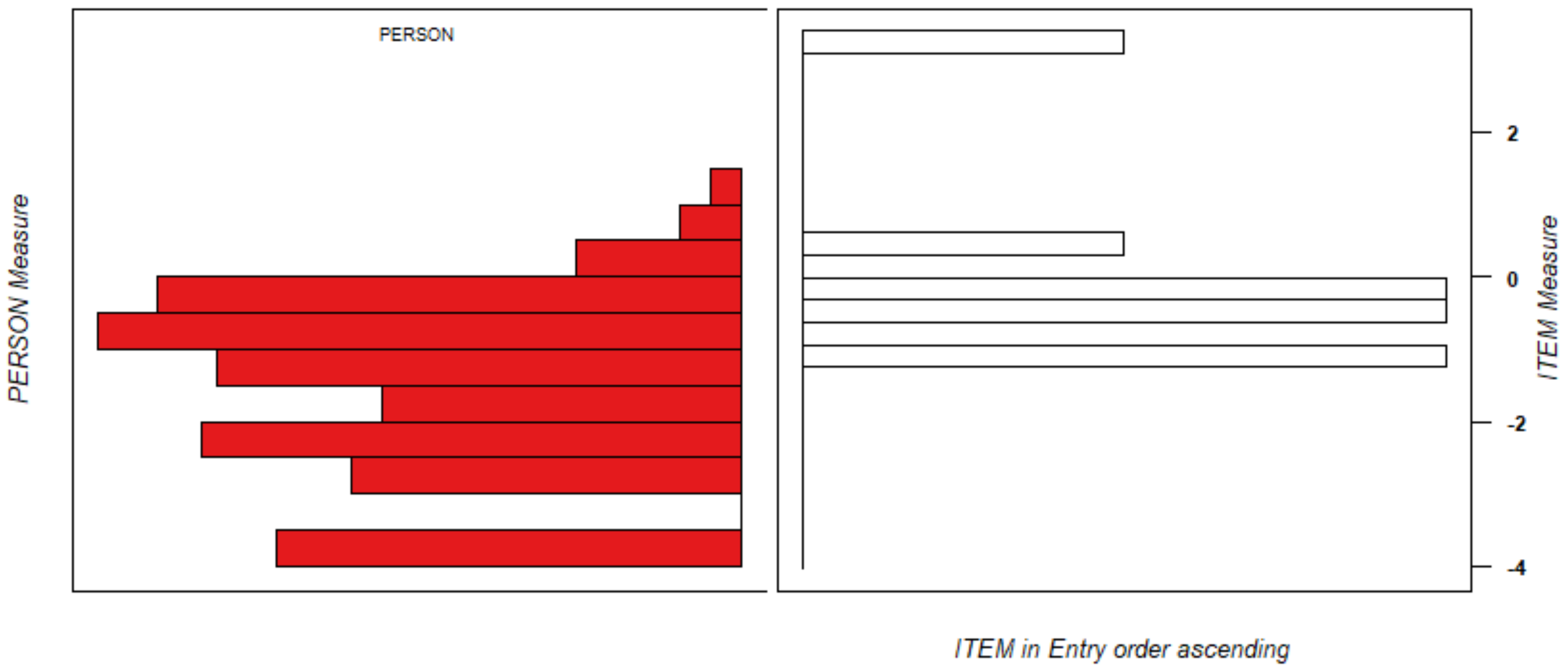

Scale: Muscles and Joints

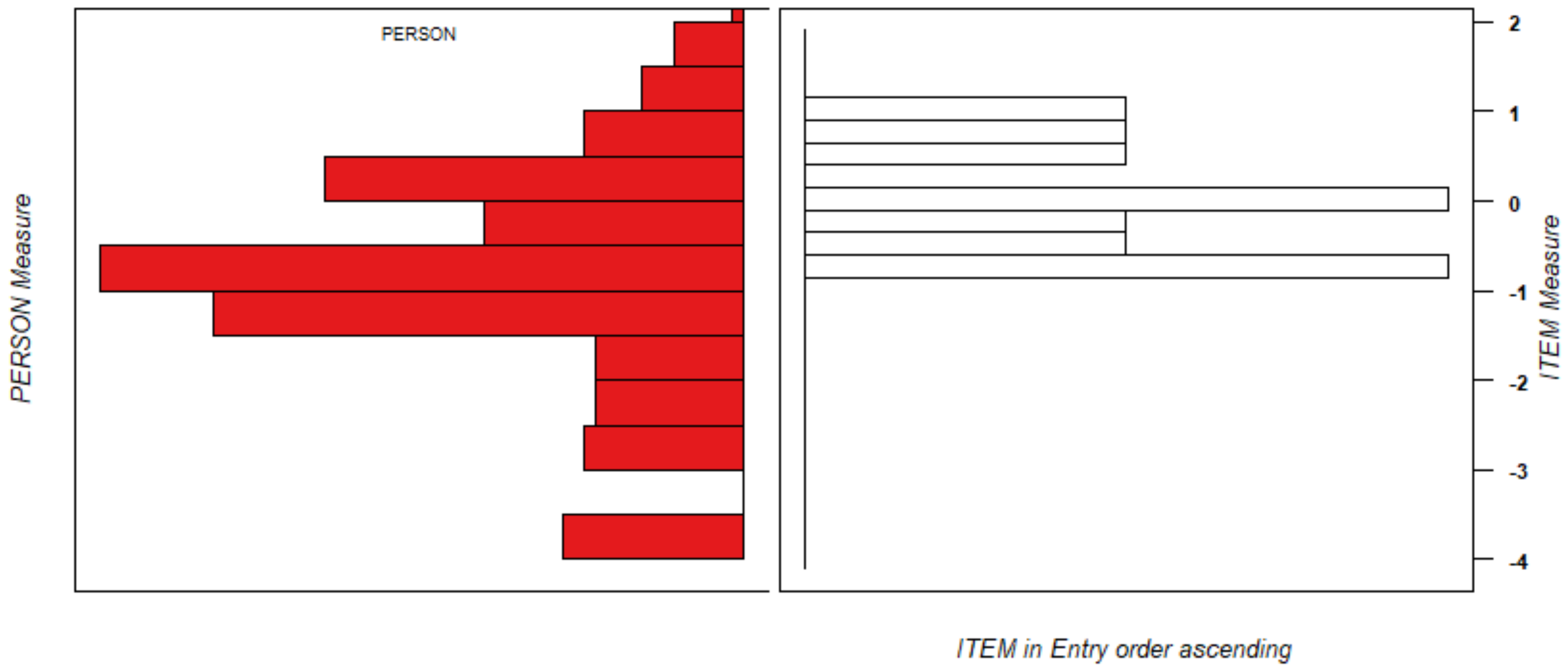

Scale: Mental Health & Wellbeing

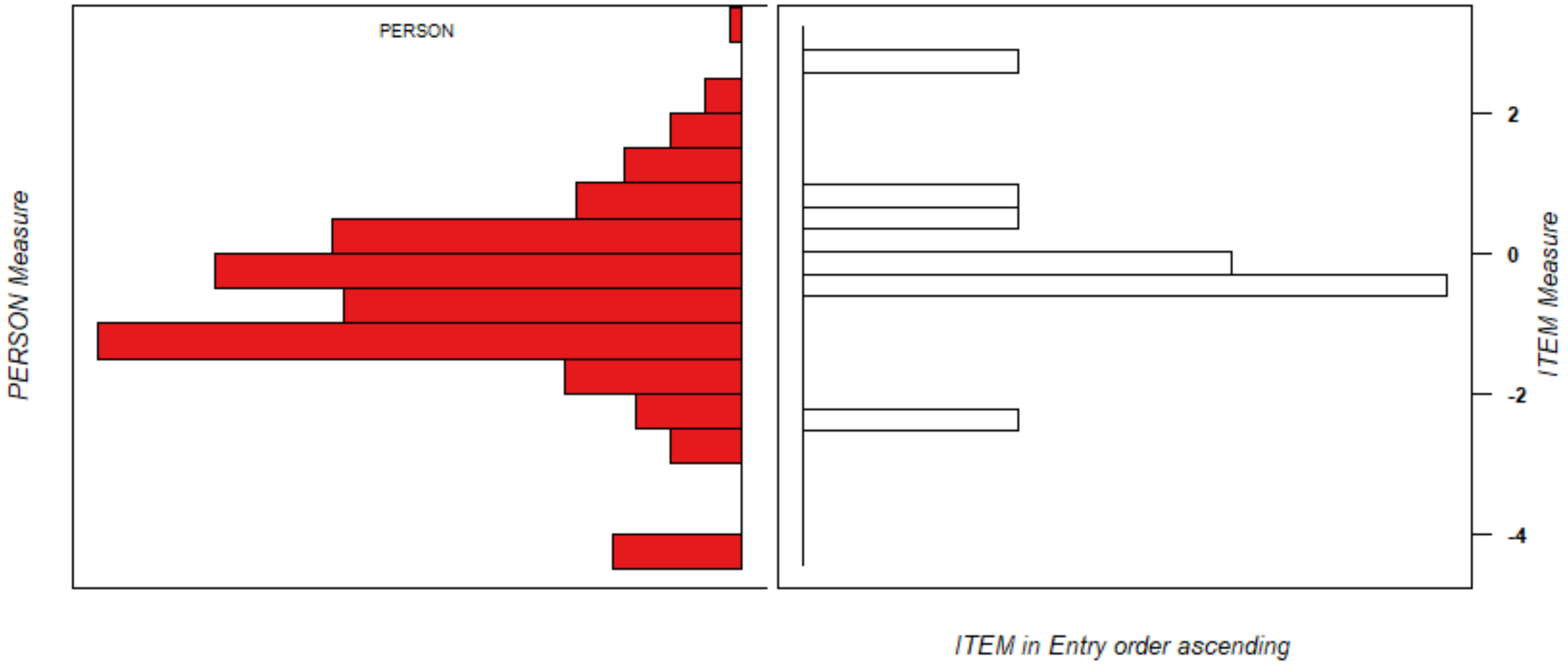

Scale: Skin and Hair

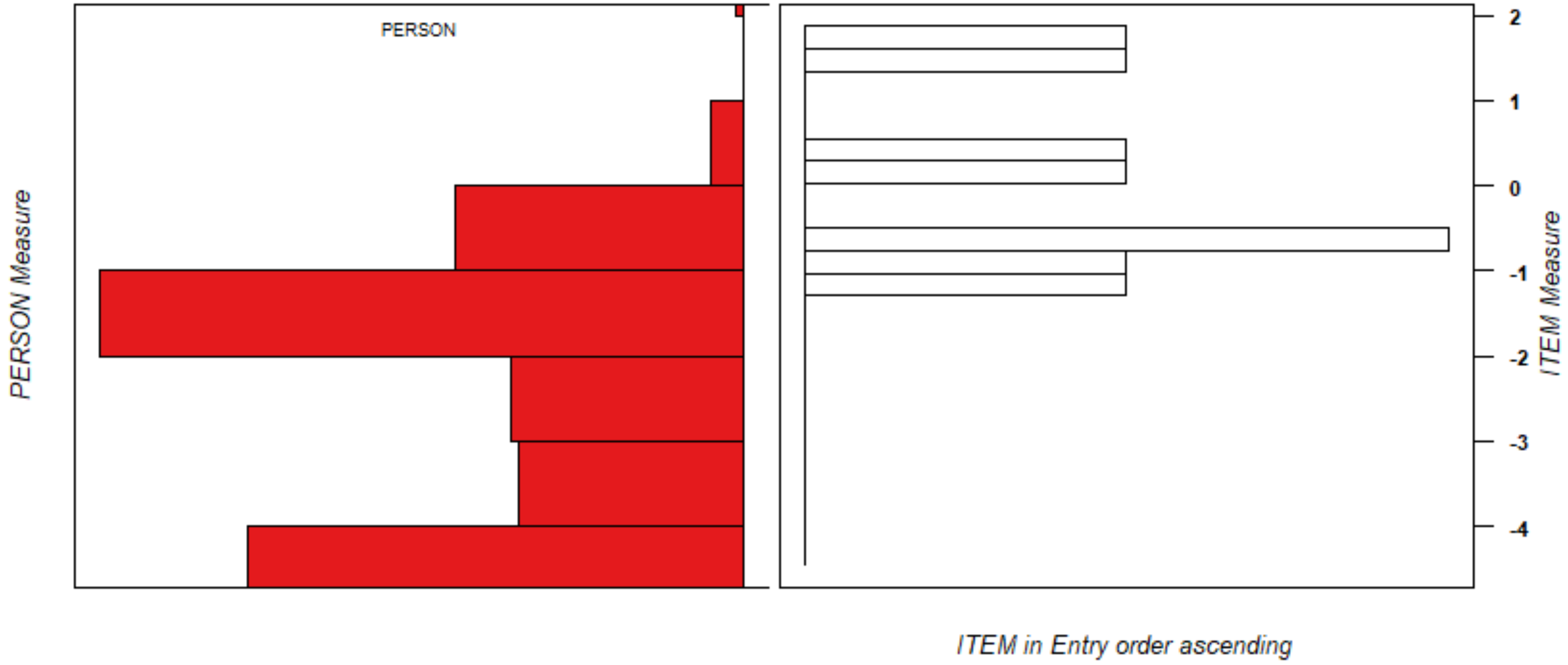

Scale: Eyes

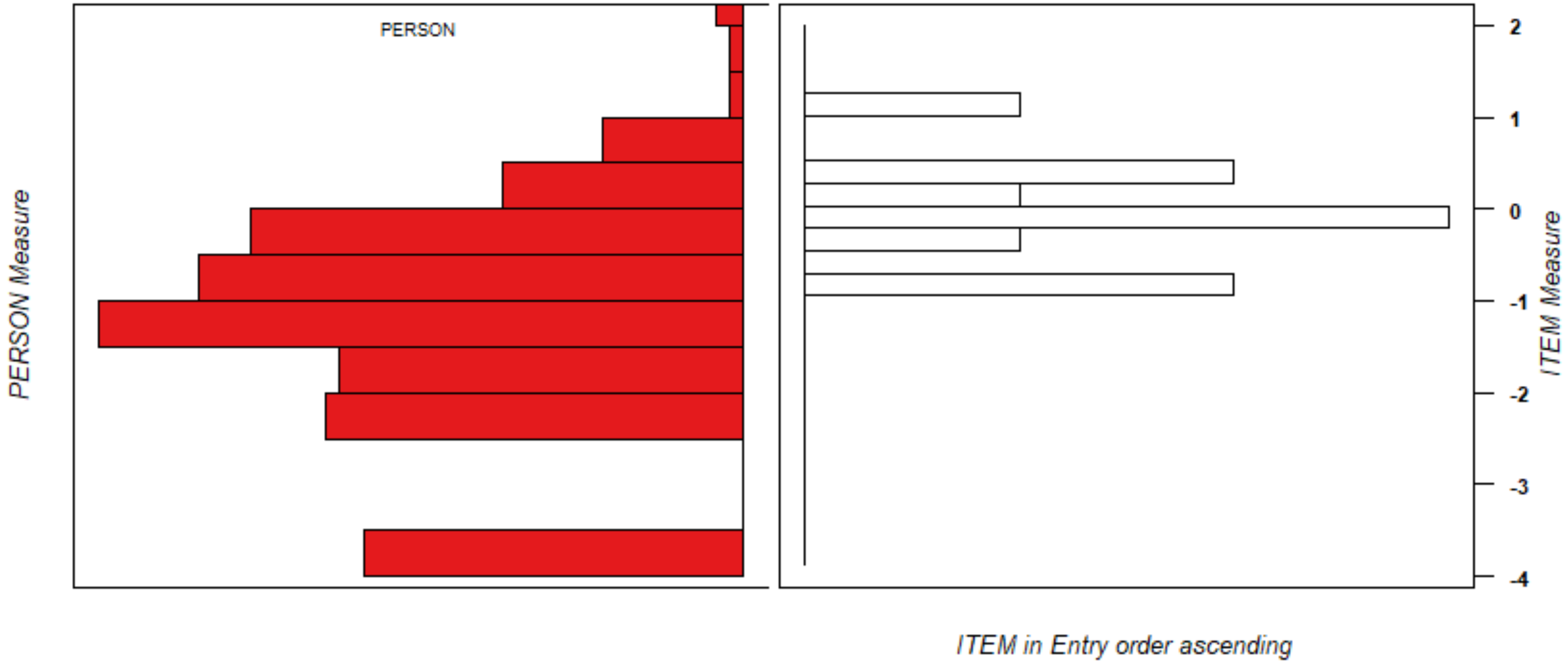
PERSON
PERSON Measure
ITEM Measure
2
1
0
-1
-2
-3
-4
ITEM in Entry order ascending

Scale: Female Reproductive and Sexual Health

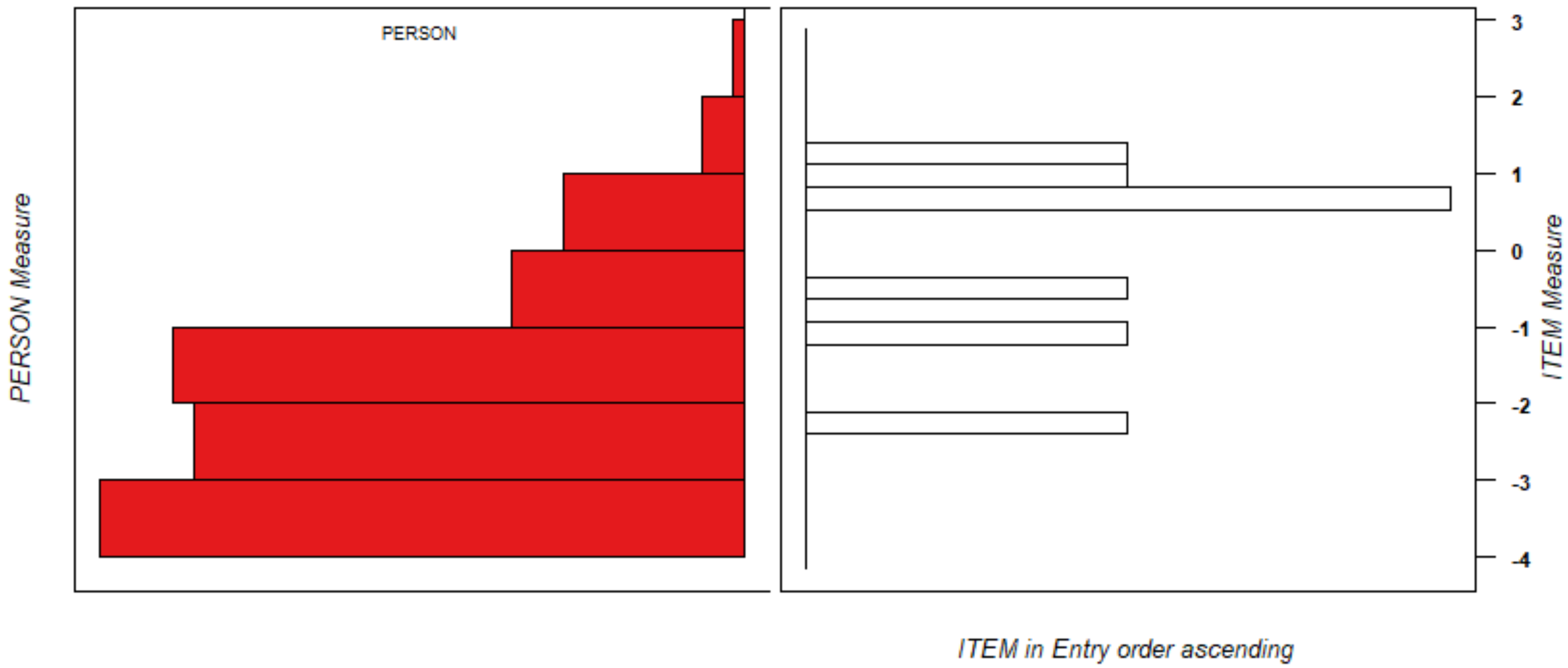

Scale: Male Reproductive and Sexual Health

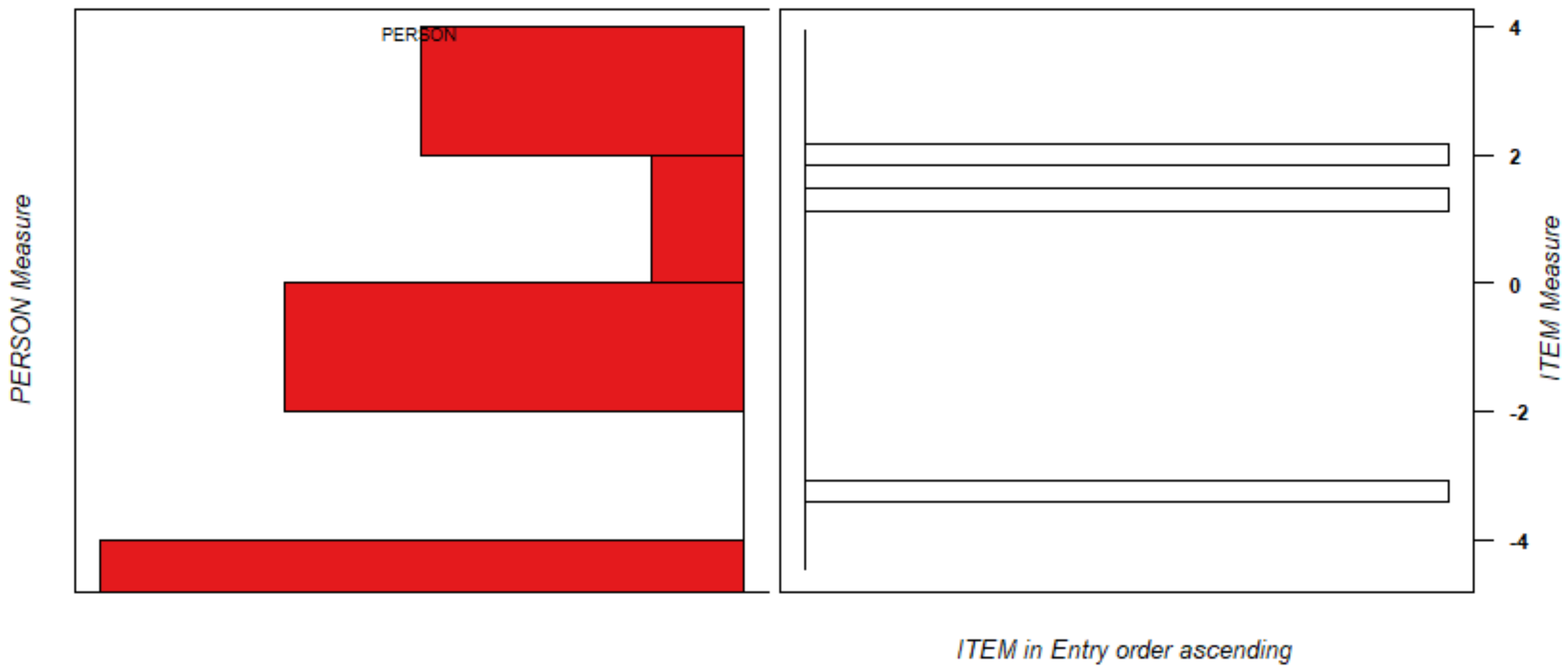

Scale: Other Symptoms

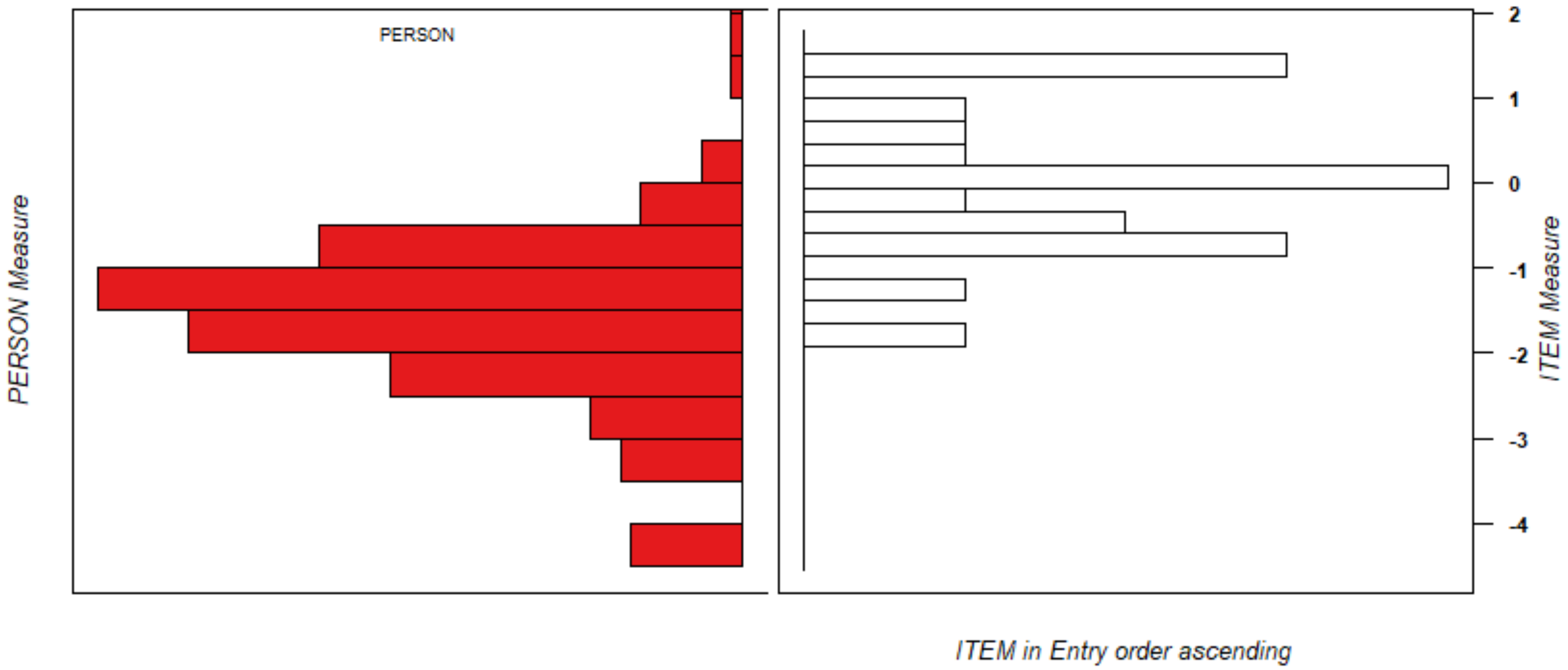

Scale: Interference

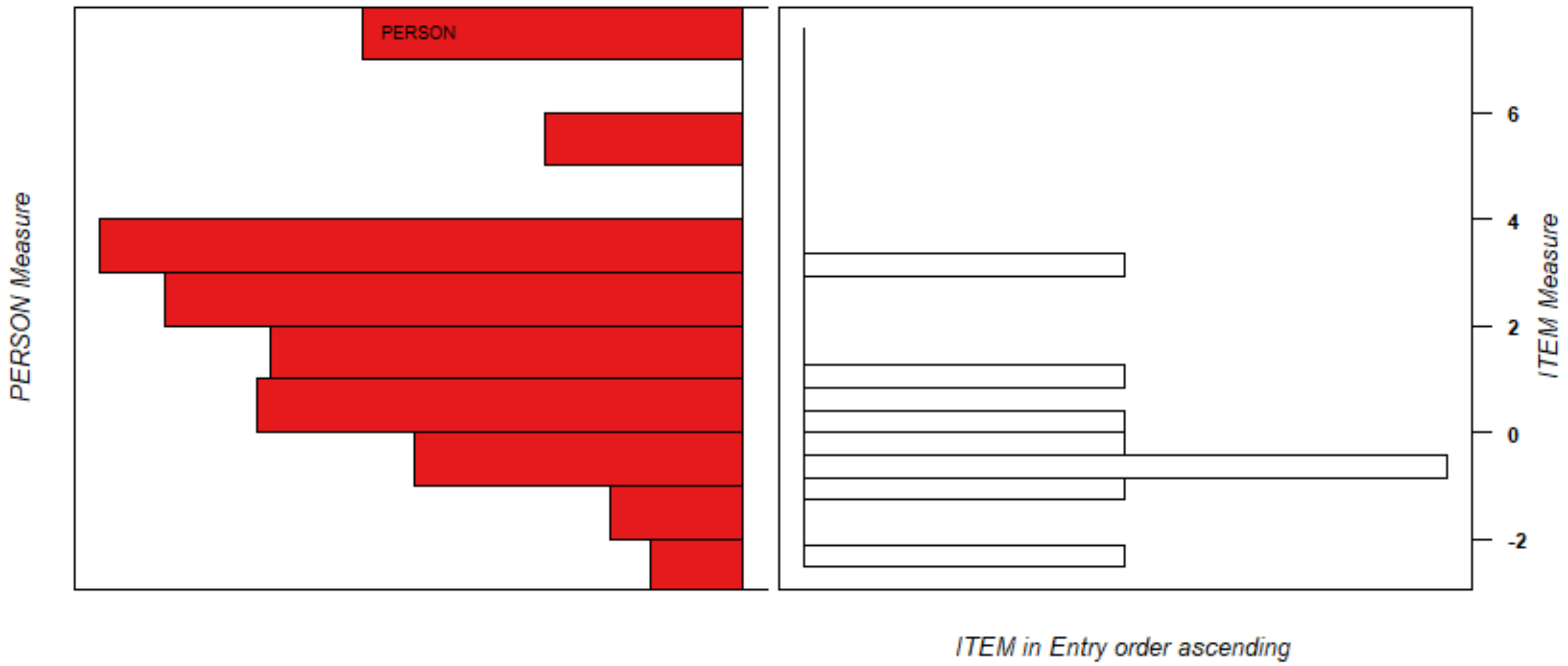

Supplement: Supplementary file 1 — Supplementary information: Additional tables S1-S4, files S1 and S2, and figures S1 and S2 [file hugs070230.ww.pdf]
